# Characterization of the fungal genus *Sphaerellopsis* associated with rust fungi: species diversity, host-specificity, biogeography, and *in-vitro* mycoparasitic events of *S. macroconidialis* on the southern corn rust, *Puccinia polysora*

**DOI:** 10.1186/s43008-024-00145-w

**Published:** 2024-07-03

**Authors:** Paula Andrea Gómez-Zapata, Jorge Ronny Díaz-Valderrama, Samira Fatemi, Cristhian Orlando Ruiz-Castro, M. Catherine Aime

**Affiliations:** 1 Department of Botany and Plant Pathology, Purdue University, West Lafayette, IN USA; 2 Grupo de Investigación en Fitopatología y Micología, Instituto de Investigación para el Desarrollo Sustentable de Ceja de Selva, Universidad Nacional Toribio Rodríguez de Amazonas, Chachapoyas, Amazonas, Peru

**Keywords:** Fungarium specimens, Leptosphaeriaceae, Mycoparasites, Natural enemies, 1 New Taxon, Pucciniales, Uredinales

## Abstract

*Sphaerellopsis* species are putative hyperparasites of rust fungi and may be promising biological control agents (BCA) of rust diseases. However, few detailed studies limit potential BCA development in *Sphaerellopsis*. Here, we explored the biogeography, host-specificity, and species diversity of *Sphaerellopsis* and examined the early infection stage of one species, *S. macroconidialis,* to infer its trophic status. We randomly screened 5,621 rust specimens spanning 99 genera at the Arthur Fungarium for the presence of *Sphaerellopsis*. We identified 199 rust specimens infected with *Sphaerellopsis* species on which we conducted morphological and multi-locus phylogenetic analyses. Five *Sphaerellopsis* species were recovered, infecting a total of 122 rust species in 18 genera from 34 countries. *Sphaerellopsis melampsorinearum* sp. nov. is described as a new species based on molecular phylogenetic data and morphological features of the sexual and asexual morphs. *Sphaerellopsis paraphysata* was the most commonly encountered species, found on 77 rust specimens, followed by *Sphaerellopsis macroconidialis* on 56 and *S. melampsorinearum* on 55 examined specimens. The type species, *Sphaerellopsis filum*, was found on 12 rust specimens and *Sphaerellopsis hakeae* on a single specimen. We also recovered and documented for the first time, the sexual morph of *S. macroconidialis*, from a specimen collected in Brazil. Our data indicate that *Sphaerellopsis* species are not host specific and furthermore that most species are cosmopolitan in distribution. However, *S. paraphysata* is more abundant in the tropics, and *S. hakeae* may be restricted to Australia. Finally, we confirm the mycoparasitic strategy of *S. macroconidialis* through *in-vitro* interaction tests with the urediniospores of *Puccinia polysora*. Shortly after germination, hyphae of *S. macroconidialis* began growing along the germ tubes of *P. polysora* and coiling around them. After 12 days of co-cultivation, turgor loss was evident in the germ tubes of *P. polysora*, and appressorium-like structures had formed on urediniospores. The interaction studies indicate that *Sphaerellopsis* species may be more effective as a BCA during the initial stages of rust establishment.

**Supplementary Information:**

The online version contains supplementary material available at 10.1186/s43008-024-00145-w.

## Introduction

*Sphaerellopsis* Cooke (Leptosphaeriaceae, Ascomycota) is the most commonly reported fungal genus associated with rust fungi (Pucciniales, Basidiomycota). *Sphaerellopsis* species have been reported on 369 rust species and 30 genera in more than 50 countries across the globe (Kranz and Brandenburger 1981). The fungus is usually described as solitary to gregarious spherical black pycnidia that develop on sori and, thus, presumably infect rust spores and prevent their dispersion (Eriksson 1966). These black pycnidia are typically found on uredinia, the spore stage most frequently associated with severe rust disease epidemics and long-distance dispersal across continents. However, pycnidia have also been found in association with other rust spore stages. Due to the intimate association of *Sphaerellopsis* with rust fungi, this fungal genus is tentatively considered a potential biological control agent (BCA) of rust fungi, many of which cause devastating disease epidemics and yield losses worldwide (Chen et al. 2002; Kolmer et al. 2009; Lidwell-Durnin and Lapthorn 2020). Nevertheless, characterization studies of *Sphaerellopsis* are scarce, which limits its use in applied biological control research.

In 1815, the type species *Sphaerellopsis filum* was initially described as *Sphaeria filum* by Bivona-Bernadi on rusts infecting *Convolvulus sepium* and *Populus nigra* in Sicily (Bivona-Bernardi 1815). Fries transferred the species to *Phoma* as * Phoma filum* in 1823 (Fries 1823). Later, Castagne erected the genus *Darluca* and treated *Sphaeria filum* as a synonym of *Darluca vagans* (Castagne 1851). However, in 1966 Eriksson considered the epithet “*vagans*” superfluous and prioritized “*filum*” over “*vagans*” (Eriksson 1966). In 1908, Spegazzini considered *Eudarluca caricis* the teleomorph of *Darluca filum* (Spegazzini 1908). Later in 1951, Keener proved the connection between these two genera experimentally (Keener 1951). Yuan et al. 1998 confirmed this connection by obtaining the asexual morph from the teleomorph in culture studies (Yuan et al. 1998). Lastly, in 1977, Sutton transferred *Darluca filum* to the genus *Sphaerellopsis* as *S. filum* (Sutton 1977). Species of *Sphaerellopsis * are commonly found in its asexual state, and the sexual morph is rarely observed. Although *Sphaerellopsis* and *Eudarluca* are now known to be congeneric, it is still uncertain which *Sphaerellopsis* species is conspecific with *Eudarluca caricis*.

Although most scientific publications posit *Sphaerellopsis* as a mycoparasite of rust fungi, its relationship with these plant pathogens is still poorly understood. While there is some evidence of direct interaction between *S. filum* and several rust species, the nature of the interaction has not been consistently described and may vary among *S. filum* strains. For instance, some researchers argue that *S. filum* can colonize rust spores by penetrating nonspecialized hyphae and disrupting cytoplasm (Carling, D.E. Brown, M.F. Millikan, 1976; Płachecka 2005; Sappin-Trouffy 1896; Whelan et al. 1997). However, other studies report no evident cytoplasmic disruptions of rust spores when *S. filum* is present (D’Oliveira, 1941; Hulea 1939). In vitro assays demonstrated hyphal growth and conidioma development of *S. filum* when cultured with intact or ruptured rust spores (Rambo and Bean 1970). However, changes in fungal growth rate do not necessarily demonstrate that *S. filum* can infect rust fungi. Similarly, lab and field experiments have shown a significant reduction in rust infection when *S. filum* is present (Black 2012; Gordon and Pfender 2012; Yuan and Han 2000), but these conclusions are contradicted by other studies (Yuan et al. 1999). In recent years, through phylogenetic analyses, several isolates determined as  *S. filum* have turned out to be incorrectly placed in the genus *Sphaerellopsis* (Trakunyingcharoen et al. 2014). Thus, new genera were created, and new species within *Sphaerellopsis* were introduced. Hence, the previous interaction tests of *S. filum* with rust fungi remain unanswered, as the *Sphaerellopsis* specimens used in those studies may represent different species, or even belong to other genera.

Based on morphology and DNA sequence data, there are currently seven accepted *Sphaerellopsis* species. Five species are reported as mycoparasites of rust fungi: *Sphaerellopsis anomala*, *S. filum*, *S. hakeae*, *S. macroconidialis*, and *S. paraphysata* (Crous et al. 2016; Nag Raj 1993; Trakunyingcharoen et al. 2014); and two are considered saprobic: *S. artemisiae* and *S. isthmospora* (Doilom et al. 2021; Phookamsak et al. 2019). Although *S. hakeae* and *S. paraphysata* were reported to be associated with rust sori and plant tissue (Crous et al. 2016, 2018), it is unclear if the association with the host plant is parasitic or saprobic. Furthermore, it has not been proven that all *Sphaerellopsis* species associated with rust fungi are mycoparasites. Therefore, while significant improvements have been made to the taxonomy of *Sphaerellopsis* (Trakunyingcharoen et al. 2014), the parasitic relationship between its members with rust fungi remain undetermined.

Among the five *Sphaerellopsis* species known to associate with *Pucciniales*, *S. paraphysata* is the only one confirmed to have a mycoparasitic strategy. Secondary metabolites obtained from *S. paraphysata* disrupted the urediniospores cell wall of *Puccinia substriata*, leading to cellular component leakage (Ashmitha Sri et al. 2020). Inoculation of the conidia of *S. paraphysata* on the uredinia of *P. substriata* reduced rust spore germination by up to 76% (Anandakumar et al. 2019). In addition, the rust disease severity of the rust was 13% when *S. paraphysata* was present compared to the control of 86% (Anandakumar et al. 2019). However, because the species was also found in plant tissue (Crous et al. 2018), further studies are needed to discard a plant pathogenic strategy for *S. paraphysata* which would likely negate its application as a potential BCA for rust fungi.

In addition to interaction studies between members of *Sphaerellopsis* and rust fungi, other ecological studies are essential to characterize the genus and determine if any species could be suitable as a BCA of rusts. For example, knowledge of a natural enemy’s host range and geographic distribution is crucial for environmental risk assessments to prevent releasing new diseases. Furthermore, host-specificity studies can help clarify whether/which *Sphaerellopsis* species are generalists or host-specific on rust species or genera. Nevertheless, biogeography, species diversity, and the host-specificity of *Sphaerellopsis* are unknown due to the few records of the currently accepted species. Most of these records are primarily from temperate regions (Ashmitha Sri et al. 2020; Crous et al. 2016; Trakunyingcharoen et al. 2014) with limited records from the tropics.

Because most *Sphaerellopsis* species are associated with rust fungi, we adopted a strategy of screening vouchered rust specimens for the presence of incidentally co-collected *Sphaerellopsis* species. The Arthur Fungarium (PUR), housed at Purdue University, is one of the world’s largest collections of rust fungi. It holds approximately 160,000 specimens of 5,000 species collected across a broad geographic distribution and timeline, and it is one of the most diverse collections, with 132 rust genera in 14 families in the world (Purdue Herbaria 2022) with especially rich holdings (ca. 50%) of specimens from the Americas. Therefore, the present study had two aims: (1) to augment distribution and host data on the fungal genus *Sphaerellopsis* by screening PUR collections, with an emphasis on those originating from the Americas, and evaluating these for signals of host-specificity, and (2) to elucidate the strategy of *S. macroconidialis* when interacting with rust fungi, using the urediniospores of the southern corn rust, caused by *Puccinia polysora*, as a model system.

## Methods

### Collected samples

We collected black fruiting bodies of *Sphaerellopsis* from preserved rust specimens in the Arthur Fungarium (PUR) supplemented with newly collected material in Peru in 2019 and Puerto Rico in 2018. Rust specimens at PUR are stored in folders sorted by rust species in host plant families and geographic regions. When collecting *Sphaerellopsis* samples at PUR, we randomly screened these rust specimens by selecting the top, middle, and bottom specimens from the Americas shelf in each rust species folder. When collecting *Sphaerellopsis* samples from other geographic regions, we randomly selected one rust specimen per folder. Although we screened rust specimens collected across the globe, the Americas was our preferred geographic region in an effort to close this information gap. We screened each rust-infected leaf of every rust specimen under a stereoscope Olympus Model SZ2-ILST (Tokyo, Japan) and screened for visible signs of *Sphaerellopsis*-type fruiting bodies developing on the sori. Only specimens fruiting exclusively on rust sori but not on surrounding host tissue were removed for further analyses, as the ability to also fruit on host plant tissues would indicate a non-rust-specific pathogen. Then, we removed one *Sphaerellopsis*-infected sorus with a sterile razor blade per rust specimen. A new blade was used per each specimen to prevent cross-contamination. Each infected sorus was placed in a microcentrifuge tube labeled with the PUR barcode of the rust specimen and a serial number.

When collecting *Sphaerellopsis* specimens in the field, we first collected rust-infected plant leaves. Then, we looked for black fruiting bodies developing on sori under a stereoscope. If *Sphaerellopsis* was present, we isolated it by cutting a piece of the plant tissue containing both the sorus and *Sphaerellopsis* with a razor blade. Then, the plant tissue was sterilized with 1/10 dilution chlorine bleach for one minute and washed three times with sterile water. The piece of plant tissue was inoculated onto Petri dishes containing potato dextrose agar (PDA) and 50 mg/mL chloramphenicol. Petri dishes were shipped to the Aime Lab at Purdue University for further processing. Once the Petri dishes arrived at the Aime lab, we subcultured them until axenic cultures were achieved on PDA and 2% malt extract agar (MEA) with 50 mg/mL chloramphenicol. Isolates were stored long-term on PDA slants at 4 °C and in 15% (v/v) glycerol at -80 °C. Finally, we pressed, dried, and vouchered the collected rust specimens at PUR.

In total, we screened 5,621 *Pucciniales* collections for the presence of *Sphaerellopsis* (Supplementary Table 1). The following data were recorded for each *Pucciniales* specimen that was found to be co-infected with *Sphaerellopsis*: PUR accession number, rust species name, country of origin, year of collection, host plant family, genus, and species, and geolocation (Supplementary Table 2). Finally, we took macro- and microphotographs of some of the collected *Sphaerellopsis* samples with an Olympus SC30 camera and image software Olympus cellSens entry version 1.14 under a stereoscope Olympus Model SZ2-ILST and a compound microscope Olympus BH2-RFCA at PUR. Measurements of fungal structures were made using cellSens Standard 1.18 Imaging Software (Olympus).

### Identification and species concept

The collected *Sphaerellopsis* samples were identified using an integrated species concept, based on morphological characters and phylogenetic analyses (Aime et al. 2021). Original descriptions of the currently accepted *Sphaerellopsis* species were used as references for morphological comparison (Cooke M.C., 1883; Crous et al. 2016; Doilom et al. 2021; Nag Raj 1993; Phookamsak et al. 2019; Trakunyingcharoen et al. 2014).

### DNA isolation and PCR amplification

The genomic DNA of each potential *Sphaerellopsis*, collected during the screening, was extracted using the EZNA HP Fungal DNA kit (Omega Bio-Tek, Norcross, Georgia), following the manufacturer’s instructions and modifying only the incubation time in the third step. Instead of 30 min, we incubated the samples overnight to ensure complete lysis of cells in the suspension. We selected the following loci for amplification: the internal transcribed spacer (ITS) and the large subunit (LSU) of the ribosomal DNA repeat, the translation elongation factor 1-α (*tef1*) and the RNA polymerase II second largest subunit (*rpb2*). Because most of our *Sphaerellopsis* specimens were derived from fungarium collections and thus culturing was not possible, we designed specific ITS and LSU primers for amplification of these loci (Table 1). For this, we downloaded all ITS and LSU sequences of verified *Sphaerellopsis* species (Trakunyingcharoen et al. 2014) from GenBank. We also downloaded sequences of several rust species and of ubiquitous fungal species usually found in dead plant material. Multiple alignments were conducted using MUSCLE version 3.7 (Edgar 2004) in MEGA7 (Kumar et al. 2016). Conserved regions were searched for both loci in *Sphaerellopsis* sequences, excluding rusts and other fungal sequences. We selected primers that amplify approximately 250 bp in length for ITS amplification and between 600 and 700 bp for LSU amplification. Finally, we performed a BLASTn database search using our selected primers as the query to confirm that the greatest matching hits were *Sphaerellopsis* sequences. Amplification for each locus was conducted with these new, and previously published (Table 1) primers in 25-µl PCR reactions on a Mastercycler ep gradient Thermal Cycler (Eppendorf model #5341, Hauppauge, New York) that consisted of 12.5 µl of 2× MyTaq Mix (Bioline, Swedesboro, New Jersey), 1.25 µl of each 10 µM primer, and 10 µl of either 1/10 or 1/5 diluted DNA extract. Amplifications of rDNA, *tef1 *and *rpb2* loci were run under the following conditions: initial denaturation at 94 °C for 5 min (95 °C for *rpb2*/96°C for 2 min for *tef1*); followed by 40 cycles of denaturation (45 cycles for ITS) at 94 °C for 30 Sect. (95 °C for *rpb2*), annealing at 51.8 °C for 45 s for ITS/54°C for 45 s for LSU/56°C for 30 s for *tef1*/55°C for 45 s for *rpb2,* and elongation at 72 °C for 45 s (1 min for LSU and 30 s for *tef1*); and final extension at 72 °C for 7 min.

**Table 1:** Primers for PCR amplification and sequencing used in this study

Gene	Primer name	Orientation	Sequences (5’ to 3’)	Reference
ITS	EudITS2F	F	AACTTTCAACAACGGATCTCTTGGT	This study
	EudITS4R	R	ATGCTTAAGTTCAGCGGGTA	This study
	EuSP_ITS_R2	R	ATGTGCYRMGMTYCAGGC	This study
LSU	Spha_28sf1	F	GAGTGAAGCGGCAACAGCTC	This study
	Spha_28sr1	R	CGATTTGCACGTCAGAACCGC	This study
tef1	EF1-728 F	F	CATCGAGAAGTTCGAGAAGG	Carbone and Kohn 1999
	EF1-986R	R	TACTTGAAGGAACCCTTACC	Carbone and Kohn 1999
rpb2	RPB2-5F2	F	GGGGWGAYCAGAAGAAGGC	Sung et al. 2007
	fRPB2-7CR	R	CCCATRGCTTGYTTRCCCAT	Liu et al. 1999

### Electrophoresis and sequencing

We ran the PCR products in 1% agarose and stained them with GelRed (RGB4102, Phoenix Research Products) for 35 min at 110 V in a Bio-Rad electrophoresis tank to visualize PCR products. PCR products of samples that showed bands were sent to Genewiz (South Plainfield, New Jersey) for purification and subsequent sequencing in both directions with the amplification primers (Table 1). Raw sequence reads were edited manually and assembled using Sequencher version 5.2.3 (Gene Codes Co., Ann Arbor, Michigan).

### Sequence alignment and phylogenetic trees

The edited sequences were blasted against the NCBI GenBank nucleotide database (http://ncbi.nlm.nih.gov/blast/Blast.cgi) to confirm placement in *Sphaerellopsis*. To construct datasets, we downloaded publicly available DNA sequences of *Sphaerellopsis* species as references for our phylogenetic analyses; *Alternaria consortialis* was chosen as the outgroup (Table 2). Sequences were aligned using MUSCLE version 3.7 (Edgar 2004) in MEGA7 (Kumar et al. 2016). Then, the aligned sequences were trimmed using trimAl version 1.2 (Capella-Gutiérrez et al. 2009) with a minimum percentage of positions to conserve [0-100]: 50; and gap threshold, the fraction of positions without gaps in a column [0–1]: 0.6. We performed maximum likelihood (ML) inference using IQ-TREE (Minh et al. 2020) under partitioned models (Chernomor et al. 2016) and selected the best nucleotide substitution model under Akaike’s information criterion corrected for small sample size (AICc) using ModelFinder (Kalyaanamoorthy et al. 2017). An ultrafast bootstrap analysis was implemented with 1,000 replicates (Hoang et al. 2018). The “-bnni” option was used to reduce the risk of overestimating branch supports with UFBoot due to severe model violations. Finally, phylogenetic reconstructions with bootstrap values were visualized in FigTree version 1.4.3 (http://tree.bio.ed.ac.uk/software/figtree/) and colored in Inkscape (https://inkscape.org).

**Table 2:** Reference sequences used in phylogenetic analyses

NCBI Reference	Species Name	Gene region	Reference
MH855147.1	*Alternaria consortialis*	ITS	Woudenberg et al. (2013)
MT957065.1	*S. artemisiae*	ITS	Doilom et al. (2021)
NR_171717.1	*S. filum*	ITS	Trakunyingcharoen et al. (2014)
AY607011	*S. filum*	ITS	Liesebach and Zaspel (2004)
AY607012	*S. filum*	ITS	Liesebach and Zaspel (2004)
AY607013	*S. filum*	ITS	Liesebach and Zaspel (2004)
NR_155859.1	*S. hakeae*	ITS	Crous et al. (2016)
MK387925.1	*S. isthmospora*	ITS	Phookamsak et al. (2019)
KP170659.1	*S. macroconidialis*	ITS	Trakunyingcharoen et al. (2014)
AY607023	*S. macroconidialis*	ITS	Liesebach and Zaspel (2004)
AY607022	*S. macroconidialis*	ITS	Liesebach and Zaspel (2004)
NR_137956.1	*S. paraphysata*	ITS	Trakunyingcharoen et al. (2014)
KP170661.1	*S. paraphysata*	ITS	Trakunyingcharoen et al. (2014)
AY607015	*Sphaerellopsis* sp.	ITS	Liesebach and Zaspel (2004)
AY607014	*Sphaerellopsis* sp.	ITS	Liesebach and Zaspel (2004)
AY587134	*Sphaerellopsis* sp.	ITS	Nischwitz et al. (2005)
MH866597.1	*A. consortialis*	LSU	Woudenberg et al. (2013)
NG_088168.1	*S. artemisiae*	LSU	Doilom et al. (2021)
NG_067290.1	*S. filum*	LSU	Trakunyingcharoen et al. (2014)
KY173555.1	*S. hakeae*	LSU	Crous et al. (2016)
MK387963.1	*S. isthmospora*	LSU	Phookamsak et al. (2019)
KP170727.1	*S. macroconidialis*	LSU	Trakunyingcharoen et al. (2014)
NG_067291.1	*S. paraphysata*	LSU	Trakunyingcharoen et al. (2014)
KC584742.1	*A. consortialis*	*tef1*	Woudenberg et al. (2013)
KP170684.1	*S. macroconidialis*	*tef1*	Trakunyingcharoen et al. (2014)
KP170685.1	*S. paraphysata*	*tef1*	Trakunyingcharoen et al. (2014)
KC584482.1	*A. consortialis*	*rpb2*	Woudenberg et al. (2013)
MH108009.1	*S. paraphysata*	*rpb2*	Crous et al. (2018)

### Geographical distribution

The localities of *Sphaerellopsis* specimens with successfully amplified gene regions were used to build a geographic map. We used the GPS coordinates of each of these specimens when present. Otherwise, we generated approximated coordinates according to the locality description following a geocoding Python Script in the GitHub repository (Lynn 2017). We plotted the geographic data of each specimen on a map and colored each point by the clades formed in the multi-locus phylogenetic tree using the package Geopandas in Python (Jordahl 2014).

### Interaction experiments between conidia of *Sphaerellopsis macroconidialis* and urediniospores of *Puccinia polysora*

*Puccinia polysora* was the host from which the strain SP28 of *S. macroconidialis*, used in this study, was collected. *Puccinia polysora* is an agriculturally important fungus that causes the destructive disease Southern rust of corn (Sun et al. 2021).

### Collection and identification of urediniospores of *Puccinia polysora from* maize crops

In the summer of 2021, maize leaves infected with *P. polysora* were harvested from field-grown maize plants at the Southwest Purdue Agricultural Center, Indiana, USA, and brought to the Aime Lab. Urediniospores were collected using a mini cyclone spore collector (Tallgrass Solutions, INC; Manhattan, KS) and stored in gelatin capsules at -80 °C until further use. To confirm the identification of the rust, we amplified the LSU region using the primers of Aime (2006) and the methodologies of Aime et al. (2018) and Aime and McTaggart (2020). We amplified the LSU as it has been shown to be the most informative gene for rust species identification (Aime et al. 2017). The resulting DNA fragment was blasted against the NCBI and the Rust HUBB (Kaishain et al. 2024) databases to confirm identity.

### Cultivation of corn plants in the greenhouse and installation of humidity chamber for inoculations

Healthy corn plants (P0574AM™) were cultivated in the greenhouse facility at Lily Hall of Life Sciences, Purdue University. We planted seven 3-gallon pots with two corn seeds per pot. Following germination, we removed the weaker seedling leaving one plant per pot. Plants were maintained at a temperature range between 24 and 30 °C and watered and fertilized as needed. Next, we installed a humidity chamber for rust inoculation in the same room where plants were growing. This chamber consisted of a simple cubic structure (30 cm^3^) made of PVC pipes and covered with a white four mil plastic sheeting. A door was installed on the chamber for easy access and manipulation of the corn plants once these were inside. A PVC pipe (2 cm diam. and 20 cm length) was also inserted in one side of the chamber to connect a 2.2 L humidifier (AquaOasis™) placed outside the chamber. Finally, we placed a hygrometer inside the chamber to track temperature and humidity.

### Rehydration of urediniospores of *P. polysora *for inoculation

Before inoculating healthy corn plants with *P. polysora*, we took the urediniospores stored at -80 °C in gel capsules and rehydrated them in two steps. First, the spores contained in gel capsules were thawed at 4 °C for 16 h. Then, the urediniospores contained in gel capsules were placed inside a humidified chamber. This chamber consisted of a sterile plastic container with a 23.5% KOH beaker as a source of water vapor. This concentration of KOH gives approx. 80% of relative humidity inside the container while avoiding water condensation (Rowell 1984). Then, we sealed the chamber with a lid and let the urediniospores rehydrate for 12 h at room temperature. Once urediniospores were rehydrated, we added them into a sterile glass vial containing 0.1% tween 20. We gently mixed the spores with the solution to resuspend them and ensure no clumps were formed.

### Inoculation of corn plants with urediniospores of *P. polysora *in the greenhouse

We used a spore inoculator (Tallgrass Solutions, Manhattan, Kansas) attached to an air compressor (California Air Tools CAT-1P060S) operating in the 2–5 psi range to inoculate healthy corn plants with urediniospores immersed in 0.1% tween 20. Each healthy corn leaf was sprayed with the spore solution at 2 cm from the leaf. Once each plant was covered entirely with the spore solution, we placed them in the humidity chamber and did not close the chamber completely to ensure air circulation. Inoculations were done in the late afternoon when temperatures were lower, which helped moisture stay longer on the leaf surface and facilitated spore germination for successful infection (Borlaug Global Rust 2017). During the infection period, temperatures were held at between 23 and 30 °C and the humidifier was continuously filled with sterile distilled water to keep relative humidity between 50 and 80%. We used 16 daylight hours and eight night hours. Under optimal conditions, we observed rust symptoms on corn leaves between 7 and 15 days after inoculation.

### Harvesting of fresh conidia of *S. macroconidialis *and urediniospores of *P. polysora *for the in-vitro interaction test

Conidia of *S. macroconidialis* SP28 from a two-week-old PDA culture were harvested for the interaction test. We added 1 mL of sterile water to the medium, then slightly agitated the petri dish to let the water mix with the conidia for about a minute. Once the water turned milky from presence of suspended conidia, we collected the conidia solution with a micropipette and transferred it to a 2mL tube. Fresh urediniospores of *P. polysora* infecting corn plants in the greenhouse were also harvested for the interaction test. We collected urediniospores from open and pulverulent sori to ensure the urediniospores were mature and ready to germinate. We gently tapped the rust-infected leaf against a 2mL tube containing 0.1% tween 20 to allow the spores to fall into it. Once the tween 20 solution turned light brown, we closed the lid. The concentration of conidia and urediniospores was measured with a hemocytometer to reach a dilution of 10^4^ spores per mL. The viability of the conidia and urediniospores was checked with Trypan Blue. We used ≥ 80% viable spores as the threshold for the interaction test. 

### In-vitro interaction between spores of *S. macroconidialis* and *P. polysora*

We poured 1 mL of 1% water agar with 50 mg/mL Chloramphenicol into small Petri dishes (50 mm diam.) to set up the interaction test. Then, we added 40 uL of the urediniospore suspension to five Petri dishes. To locate the urediniospores during the interaction test, we drew two points on each side of the bottom of each petri dish with a marker. Each Petri dish was sealed with parafilm and incubated in the dark at 25°C overnight to facilitate urediniospore germination. We observed each petri dish under a compound microscope Olympus BH2-RFCA using a 20X objective the day following inoculation. Petri dishes in which > 70% of the urediniospores germinated were kept for the next step. A minimum of three Petri dishes with > 70% urediniospore germination were used as replicates for the interaction test. Then, we added the conidia of *Sphaerellopsis*, suspended in water, to the same Petri dishes containing germinated urediniospores at a 1 mm distance from the urediniospores. Petri dishes were sealed again with parafilm and incubated for 24 h at room temperature. After 24 h of co-inoculation, we conducted daily screenings of the plates over the next 12 days. Interactions were observed under a microscope using the 20X objective without opening the lid to avoid contamination. Lids were only removed on the last day of observation to use a 40x objective and to take final pictures. Two negative controls were also used in this interaction test. The first one consisted of three Petri dishes containing urediniospores only. The second consisted of three Petri dishes containing only conidia of *S. macroconidialis*. The experiment was repeated three times.

## Results

### *Sphaerellopsis* recovery from PUR collections

We randomly screened 5,621 rust specimens in 99 rust genera, representing 5% of the total collections at PUR and 58% of the accepted rust genera (Berndt & Aime, n.d. unpublished), for the presence of *Sphaerellopsis* species that were incidentally co-collected with rust specimens (Supplementary Table 1). Of these 5,621 specimens, we collected 523 black fruiting bodies resembling the fungal genus *Sphaerellopsis* (Supplementary Table 2). Of these 523 collections, 199 were confirmed as *Sphaerellopsis* members through phylogenetic analyses and morphology (Table 3). Five *Sphaerellopsis* species were recovered, infecting 122 rust species in 18 genera from 34 countries.

**Table 3:** *Sphaerellopsis* members associated with rust fungi from PUR and identified through molecular and morphological analyses. Reference sequences in bold; NA: data not available

Taxon	Voucher number (Collection number)	Host Rust	Locality/Origin	ITS	LSU	TEF-1α	RPB2
***Alternaria consortialis***	**CBS 104.31**	—	NA	**MH855147.1**	**MH866597.1**	**KC584742.1**	**KC584482.1**
***Sphaerellopsis artemisiae***	**KUMCC 20-0202A**	*Artemisia argyi*	China	**MT957065.1**	**NG_088168.1**	—	—
***Sphaerellopsis filum***	**CBS 317.68**	*Puccinia deschampsiae*	Germany	**NR_171717.1**	**NG_067290.1**	—	—
*Sphaerellopsis filum*	N16937_SPH	*Puccinia graminis*	USA	OQ418215	—	—	—
*Sphaerellopsis filum*	PUR62890	*Puccinia coronata*	Mexico	OQ418251	OQ418161	—	—
*Sphaerellopsis filum*	PURF16864/ PUR83764	*Tranzschelia discolor*	Ecuador	OQ418260	—	—	—
*Sphaerellopsis filum*	PURF17349	*Puccinia brachypodii*	Chile	OQ418297	—	—	—
*Sphaerellopsis filum*	PURF19494	*Puccinia brachypodii*	Argentina	OQ418305	OQ418171	—	—
*Sphaerellopsis filum*	PURF3782	*Puccinia brachypodii*	Bolivia	OQ418317	—	—	—
*Sphaerellopsis filum*	PURF4112	*Puccinia recondita*	Ecuador	OQ418322	OQ418174	—	—
*Sphaerellopsis filum*	PURF4300	*Puccinia graminis*	Ecuador	OQ418323	—	—	—
*Sphaerellopsis filum*	PURF5980	*Puccinia boerhaviae*	Ecuador	OQ418329	—	—	—
*Sphaerellopsis filum*	PURN12884	*Melampsora ferrinii*	Peru	OQ418352	—	—	—
*Sphaerellopsis filum*	PURN4541a	*Puccinia coronata*	Germany	OQ418396	—	—	—
*Sphaerellopsis filum*	PURN5297	*Puccinia graminis*	Mexico	OQ418398	—	—	—
***Sphaerellopsis filum***	**s15**	*Melampsora* sp.	Germany	**AY607011**	—	—	—
***Sphaerellopsis filum***	**s27**	*Melampsora* sp.	Germany	**AY607012**	—	—	—
***Sphaerellopsis filum***	**s45**	*Melampsora* sp.	Germany	**AY607013**	—	—	—
***Sphaerellopsis hakeae***	**CPC 29566**	*Hakea* sp.	Australia	**NR_155859.1**	**KY173555.1**	—	—
*Sphaerellopsis hakeae*	PURF10892	*Uromyces ehrhartae*	Australia	OQ418277	—	—	—
***Sphaerellopsis isthmospora***	**HKAS 102225A**	*Dead branches*	China	**MK387925.1**	**MK387963.1**	—	—
***Sphaerellopsis macroconidialis***	**CBS 658.78**	*Puccinia allii*	Netherlands	**KP170659.1**	**KP170727.1**	**KP170684.1**	
*Sphaerellopsis macroconidialis*	Eud3.1	*Puccinia sorghi*	Peru	OQ418213	OQ418155	OQ743695	—
*Sphaerellopsis macroconidialis*	PUR19637	*Puccinia graminis*	Canada	OQ418219	—	—	—
*Sphaerellopsis macroconidialis*	PUR25166	*Puccinia recondita*	USA	OQ418224	—	—	—
*Sphaerellopsis macroconidialis*	PUR26871	*Puccinia fuirenicola*	Cuba	OQ418225	—	—	—
*Sphaerellopsis macroconidialis*	PUR40715	*Puccinia incondita*	USA	OQ418229	—	—	—
*Sphaerellopsis macroconidialis*	PUR41914	*Puccinia grindeliae*	USA	OQ418230	—	—	—
*Sphaerellopsis macroconidialis*	PUR55871	*Puccinia eatoniae*	USA	OQ418241	OQ418160	OQ743701	—
*Sphaerellopsis macroconidialis*	PUR60157	*Ravenelia thornberiana*	Mexico	OQ418246	—	—	—
*Sphaerellopsis macroconidialis*	PUR60992	*Puccinia thaliae*	Paraguay	OQ418248	—	—	—
*Sphaerellopsis macroconidialis*	PUR62883	*Puccinia unica*	Spain	OQ418250	—	—	—
*Sphaerellopsis macroconidialis*	PUR6299	*Ravenelia cassiaecola*	USA	OQ418252	—	—	—
*Sphaerellopsis macroconidialis*	PUR64476	*Puccinia poarum*	Mexico	OQ418254	—	—	—
*Sphaerellopsis macroconidialis*	PUR66593	*Phakopsora apoda*	Ecuador	OQ418256	—	—	—
*Sphaerellopsis macroconidialis*	PUR69289	NA	NA	OQ418258	OQ418162	OQ743702	—
*Sphaerellopsis macroconidialis*	PUR7334	*Uropyxis diphysae*	Guatemala	OQ418259	—	—	—
*Sphaerellopsis macroconidialis*	PUR8475	*Phragmidium guatemalense*	Guatemala	OQ418261	—	—	—
*Sphaerellopsis macroconidialis*	PUR87630	*Maravalia erythroxyli*	Brazil	—	OQ418164	—	—
*Sphaerellopsis macroconidialis*	PUR88221	*Puccinia recondita*	Brazil	OQ418263	—	OQ743704	—
*Sphaerellopsis macroconidialis*	PUR88382	*Uromyces silphii*	Canada	OQ418266	—	—	—
*Sphaerellopsis macroconidialis*	PUR90210	*Phakopsora coca*	Brazil	OQ418270	OQ418166	OQ743706	—
*Sphaerellopsis macroconidialis*	PURF10361	*Uromyces striatus*	Argentina	OQ418272	—	—	—
*Sphaerellopsis macroconidialis*	PURF10651	*Puccinia brachypoii var. poaememoralis*	Colombia	OQ418273	—	—	—
*Sphaerellopsis macroconidialis*	PURF10657	*Puccinia spilanthicola*	Colombia	OQ418274	—	—	—
*Sphaerellopsis macroconidialis*	PURF10865	*Puccinia recondita*	Australia	OQ418276	—	—	—
*Sphaerellopsis macroconidialis*	PURF10996	*Phakopsora lenticularis*	Venezuela	OQ418278	—	—	—
*Sphaerellopsis macroconidialis*	PURF11699	*Puccinia graminis*	Argentina	OQ418283	—	—	—
*Sphaerellopsis macroconidialis*	PURF1211	*Phakopsora compressa*	Bolivia	OQ418284	—	—	—
*Sphaerellopsis macroconidialis*	PURF1212	*Phakopsora compressa*	Bolivia	OQ418285	—	—	—
*Sphaerellopsis macroconidialis*	PURF17655	*Puccinia brachypodii*	Argentina	OQ418298	—	—	—
*Sphaerellopsis macroconidialis*	PURF17656	*Puccinia brachypodii*	Venezuela	OQ418299	—	—	—
*Sphaerellopsis macroconidialis*	PURF17814	*Puccinia polypogonis*	Brazil	OQ418300	—	—	—
*Sphaerellopsis macroconidialis*	PURF18990	*Phakopsora coca*	Brazil	OQ418303	—	—	—
*Sphaerellopsis macroconidialis*	PURF2397	*Uromyces epicampis*	Ecuador	OQ418311	OQ418172	OQ743712	—
*Sphaerellopsis macroconidialis*	PURF2408	*Uromyces pencanus*	Chile	OQ418312	—	—	—
*Sphaerellopsis macroconidialis*	PURF2409	*Uromyces pencanus*	Chile	OQ418313	—	—	—
*Sphaerellopsis macroconidialis*	PURF3797	*Puccinia brachypoii var. poaememoralis*	Ecuador	OQ418318	OQ418173	OQ743713	—
*Sphaerellopsis macroconidialis*	PURF3799	*Puccinia brachypoii var. poaememoralis*	Ecuador	OQ418319	—	—	—
*Sphaerellopsis macroconidialis*	PURF3854	*Puccinia poarum*	Brazil	OQ418320	—	—	—
*Sphaerellopsis macroconidialis*	PURF3879	*Puccinia moliniae*	Germany	OQ418321	—	—	—
*Sphaerellopsis macroconidialis*	PURF4648	*Puccinia aegopogonis*	Ecuador	OQ418324	—	—	—
*Sphaerellopsis macroconidialis*	PURF4891	*Puccinia substriata*	Bolivia	OQ418327	—	—	—
*Sphaerellopsis macroconidialis*	PURF6912	*Puccinia hydrocotyles*	Colombia	OQ418330	—	—	—
*Sphaerellopsis macroconidialis*	PURF8187	*Puccinia praedicata*	Brazil	OQ418333	—	—	—
*Sphaerellopsis macroconidialis*	PURF8347	*Puccinia wedellicola*	Brazil	OQ418336	—	—	—
*Sphaerellopsis macroconidialis*	PURF9548	*Puccinia bonariensis*	Argentina	OQ418337	OQ418177	OQ743717	—
*Sphaerellopsis macroconidialis*	PURN11506	*Puccinia persistens*	USA	OQ418345	—	—	—
*Sphaerellopsis macroconidialis*	PURN11560	*Phakopsora apoda*	Peru	OQ418346	OQ418179	OQ743721	—
*Sphaerellopsis macroconidialis*	PURN11633	*Puccinia pygmaea*	USA	OQ418347	OQ418180	OQ743722	OQ587604
*Sphaerellopsis macroconidialis*	PURN16382	*Tranzschelia mexicana*	Peru	OQ418360	OQ418188	OQ743731	—
*Sphaerellopsis macroconidialis*	PURN23084	*Puccinia sorghi*	Peru	OQ418370	OQ418194	OQ743734	OQ587609
*Sphaerellopsis macroconidialis*	PURN2544	*Uromyces iresines*	Ecuador	OQ418373	—	OQ743735	—
*Sphaerellopsis macroconidialis*	PURN3032	*Puccinia fumosa*	Mexico	OQ418375	—	—	—
*Sphaerellopsis macroconidialis*	PURN4199	*Chaconia brasiliensis*	Brazil	OQ418392	OQ418205	OQ743739	—
*Sphaerellopsis macroconidialis*	PURN4207	*Chaconia brasiliensis*	Brazil	OQ418393	—	—	—
*Sphaerellopsis macroconidialis*	PURN4452	*Chrysocelis muehlenbeckiae*	Colombia	OQ418394	OQ418206	OQ743740	—
***Sphaerellopsis macroconidialis***	**s101**	*Puccinia obscura*	Germany	**AY607023**	—	—	—
***Sphaerellopsis macroconidialis***	**s13**	*Puccinia abrupta*	Ethiopia	**AY607022**	—	—	—
*Sphaerellopsis macroconidialis*	SP28	*Puccinia sorghi*	Peru	OQ418406	OQ418211	OQ743743	OQ587610
***Sphaerellopsis melampsorinearum*** **sp. nov.**	**ATCC MYA-2847**	*Melampsora medusae*	USA	**AY587134**	—	—	—
*Sphaerellopsis melampsorinearum* sp. nov.	PUR11683	*Uromyces graminicola*	USA	OQ418217	—	—	—
*Sphaerellopsis melampsorinearum* sp. nov.	PUR2041	*Melampsora medusae*	USA	OQ418220	—	—	—
*Sphaerellopsis melampsorinearum* sp. nov.	PUR2047	*Melampsora medusae*	USA	OQ418221	—	OQ743698	—
*Sphaerellopsis melampsorinearum* sp. nov.	PUR2129	*Melampsora medusae*	USA	OQ418222	—	—	—
*Sphaerellopsis melampsorinearum *sp. nov.	PUR32274	*Puccinia proserpinacae*	USA	OQ418227	—	—	—
*Sphaerellopsis melampsorinearum* sp. nov.	PUR43744	*Coleosporium helianthi*	USA	OQ418232	—	—	—
*Sphaerellopsis melampsorinearum* sp. nov.	PUR43798	*Coleosporium helianthi*	USA	OQ418233	—	—	—
*Sphaerellopsis melampsorinearum* sp. nov.	PUR47887	*Melampsora medusae*	USA	OQ418234	—	—	—
*Sphaerellopsis melampsorinearum* sp. nov.	PUR56253	*Puccinia recondita*	USA	OQ418243	—	—	—
*Sphaerellopsis melampsorinearum* sp. nov.	PUR88233	*Coleosporium helianthi*	USA	OQ418265	—	—	—
*Sphaerellopsis melampsorinearum* sp. nov.	PUR90026	*Melampsora ferrinii*	Brazil	OQ418267	—	OQ743705	—
*Sphaerellopsis melampsorinearum* sp. nov.	PUR90183	*Melampsora larici-populina*	Brazil	OQ418269	OQ418165	—	—
*Sphaerellopsis melampsorinearum* sp. nov.	PUR90242	*Melampsora epitea*	Brazil	OQ418271	OQ418167	—	—
*Sphaerellopsis melampsorinearum* sp. nov.	PURF11501	*Puccinia thaliae*	Venezuela	OQ418281	OQ418168	—	—
*Sphaerellopsis melampsorinearum* sp. nov.	PURF14716	*Puccinia sorghi*	Ecuador	OQ418289	OQ418170	OQ743708	—
*Sphaerellopsis melampsorinearum* sp. nov.	PURF16121	*Puccinia phaenospermae*	Japan	OQ418294	—	OQ743709	—
*Sphaerellopsis melampsorinearum* sp. nov.	PURF1651	*Gerwasia holwayi*	Peru	OQ418295	—	—	—
*Sphaerellopsis melampsorinearum* sp. nov.	PURF17310	*Puccinia caricis-japonica*	Japan	OQ418296	—	—	—
*Sphaerellopsis melampsorinearum* sp. nov.	PURF3626	*Uromyces wulffiae-stenoglossae*	Trinidad	OQ418316	—	—	—
*Sphaerellopsis melampsorinearum* sp. nov.	PURF7943	*Puccinia inaequata*	Ecuador	OQ418331	—	—	—
*Sphaerellopsis melampsorinearum* sp. nov.	PURF829	*Melampsora medusae*	Bolivia	OQ418334	—	—	—
*Sphaerellopsis melampsorinearum* sp. nov.	PURF833	*Melampsora aecidioides*	Argentina	OQ418335	OQ418176	OQ743716	—
*Sphaerellopsis melampsorinearum* sp. nov.	PURN12037	*Melampsora humboldtiana*	USA	OQ418350	OQ418182	—	—
*Sphaerellopsis melampsorinearum* sp. nov.	PURN1206	*Uromyces minutus*	USA	OQ418351	—	—	—
*Sphaerellopsis melampsorinearum* sp. nov.	PURN15307(WTHC1)	*Melampsora medusae*	USA	OQ418354	OQ418183	OQ743726	OQ587607
*Sphaerellopsis melampsorinearum *sp. nov.	PURN16392	*Uromyces yurimaguensis*	Peru	OQ418361	OQ418189	—	—
*Sphaerellopsis melampsorinearum* sp. nov.	PURN16518	*Melampsora ferrinii*	USA	OQ418362	OQ418190	—	—
*Sphaerellopsis melampsorinearum* sp. nov.	PURN16527	*Melampsora medusae*	USA	OQ418363	—	—	—
*Sphaerellopsis melampsorinearum* sp. nov.	PURN21944	NA	NA	OQ418366	OQ418193	—	—
*Sphaerellopsis melampsorinearum* sp. nov.	PURN2294	*Coleosporium montanum*	USA	OQ418367	—	—	—
*Sphaerellopsis melampsorinearum* sp. nov.	PURN2303	*Coleosporium asterum*	USA	OQ418369	—	—	—
*Sphaerellopsis melampsorinearum* sp. nov.	PURN2314	*Coleosporium asterum*	USA	OQ418371	—	—	—
*Sphaerellopsis melampsorinearum* sp. nov.	PURN2448	*Melampsora epitea*	USA	OQ418372	—	—	—
*Sphaerellopsis melampsorinearum* sp. nov.	PURN3993	*Melampsora epitea*	Brazil	OQ418376	OQ418195	—	—
*Sphaerellopsis melampsorinearum* sp. nov.	PURN3995	*Melampsora ferrinii*	Argentina	OQ418377	—	—	—
*Sphaerellopsis melampsorinearum* sp. nov.	PURN3996	*Melampsora epitea*	Argentina	OQ418378	OQ418196	—	—
*Sphaerellopsis melampsorinearum* sp. nov.	PURN3997	*Melampsora epitea*	Argentina	OQ418379	OQ418197	OQ743736	—
*Sphaerellopsis melampsorinearum* sp. nov.	PURN3998	*Melampsora epitea*	Argentina	OQ418380	—	—	—
*Sphaerellopsis melampsorinearum* sp. nov.	PURN4001	*Melampsora epitea*	Argentina	OQ418381	OQ418198	—	—
*Sphaerellopsis melampsorinearum* sp. nov.	PURN4010	*Melampsora medusae*	USA	OQ418382	—	—	—
*Sphaerellopsis melampsorinearum* sp. nov.	PURN4015	*Melampsora larici-populina*	Colombia	OQ418383	OQ418199	—	—
*Sphaerellopsis melampsorinearum* sp. nov.	PURN4108	*Melampsora aecidioides*	Brazil	OQ418384	OQ418200	OQ743737	—
*Sphaerellopsis melampsorinearum* sp. nov.	PURN4109	*Melampsora larici-populina*	Brazil	OQ418385	—	—	—
*Sphaerellopsis melampsorinearum* sp. nov.	PURN4120	*Melampsora ferrinii*	Brazil	OQ418386	—	—	—
*Sphaerellopsis melampsorinearum* sp. nov.	PURN4121	*Melampsora ferrinii*	Brazil	OQ418387	OQ418201	—	—
*Sphaerellopsis melampsorinearum* sp. nov.	PURN4124	*Melampsora epitea*	Brazil	OQ418390	OQ418204	—	—
*Sphaerellopsis melampsorinearum* sp. nov.	PURN4127	*Melampsora ferrinii*	Brazil	OQ418391	—	—	—
*Sphaerellopsis melampsorinearum* sp. nov.	PURN4510	*Melampsora medusae*	USA	OQ418395	OQ418207	—	—
*Sphaerellopsis melampsorinearum* sp. nov.	PURN5424	*Melampsora medusae*	USA	OQ418399	OQ418208	OQ743741	—
Sphaerellopsis melampsorinearum sp. nov.	PURN6730	*Melampsora medusae*	USA	OQ418402	OQ418209	OQ743742	—
*Sphaerellopsis melampsorinearum *sp. nov.	PURN8265	*Melampsora epitea*	Colombia	OQ418403	—	—	—
*Sphaerellopsis melampsorinearum* sp. nov.	PURN9763	*Puccinia vernoniae-mollis*	Brazil	OQ418405	OQ418210	—	—
***Sphaerellopsis melampsorinearum*** **sp. nov.**	**s18**	*Melampsora* sp.	Germany	**AY607014**	—	—	—
***Sphaerellopsis melampsorinearum*** **sp. nov.**	**s21**	*Melampsora* sp.	Germany	**AY607015**	—	—	—
***Sphaerellopsis paraphysata***	**CBS 143579**	*Leaves of Phragmites* sp.	Australia	—	—	—	**MH108009.1**
***Sphaerellopsis paraphysata***	**CPC 21841**	—	Brazil	**NR_137956.1**	**NG_067291.1**	**KP170685.1**	—
***Sphaerellopsis paraphysata***	**CPC 23547**	*Ravenelia macowania*	South Africa	**KP170661.1**	—	—	—
*Sphaerellopsis paraphysata*	MCA7075	*Puccinia aframomi*	Cameroon	OQ418214	OQ418156	OQ743696	OQ587603
*Sphaerellopsis paraphysata*	PAZ14rust-PURN23070	NA	USA	—	OQ418157	OQ743697	—
*Sphaerellopsis paraphysata*	PP2004 (PURN11661)	*Puccinia philippinensis*	Guam	OQ418349	—	OQ743724	—
*Sphaerellopsis paraphysata*	PUR11619	*Uromyces andropogonis*	USA	OQ418216	—	—	—
*Sphaerellopsis paraphysata*	PUR15359	*Uromyces trifolii-repentis*	USA	OQ418218	—	—	—
*Sphaerellopsis paraphysata*	PUR31535	*Puccinia* sp.	USA	OQ418226	—	—	—
*Sphaerellopsis paraphysata*	PUR3364	*Pucciniastrum hydrangeae*	USA	OQ418228	—	—	—
*Sphaerellopsis paraphysata*	PUR42807	*Crossopsora hymenaeae*	Cuba	OQ418231	—	—	—
*Sphaerellopsis paraphysata*	PUR48117	*Puccinia levis*	USA	OQ418235	OQ418158	OQ743699	—
*Sphaerellopsis paraphysata*	PUR50338	*Uromyces bonariensis*	Guatemala	OQ418236	—	—	—
*Sphaerellopsis paraphysata*	PUR50994	*Uromyces* sp.	USA	OQ418237	—	—	—
*Sphaerellopsis paraphysata*	PUR51302	*Phakopsora aurea*	Honduras	OQ418238	OQ418159	OQ743700	—
*Sphaerellopsis paraphysata*	PUR52253	*Puccinia arthurella*	Trinidad	OQ418239	—	—	—
*Sphaerellopsis paraphysata*	PUR52702	*Phakopsora compressa*	Costa Rica	OQ418240	—	—	—
*Sphaerellopsis paraphysata*	PUR56162	*Puccinia marylandica*	USA	OQ418242	—	—	—
*Sphaerellopsis paraphysata*	PUR58549	*Puccinia stenotaphri*	USA	OQ418244	—	—	—
*Sphaerellopsis paraphysata*	PUR59371	*Puccinia unica*	Mexico	OQ418245	—	—	—
*Sphaerellopsis paraphysata*	PUR60362	*Puccinia subtilipes*	Honduras	OQ418247	—	—	—
*Sphaerellopsis paraphysata*	PUR62882	*Puccinia unica*	USA	OQ418249	—	—	—
*Sphaerellopsis paraphysata*	PUR64079	*Uromyces costaricensis*	Mexico	OQ418253	—	—	—
*Sphaerellopsis paraphysata*	PUR64614	*Uromyces epicampis*	Mexico	OQ418255	—	—	—
*Sphaerellopsis paraphysata*	PUR66889	*Sorataea baphiae*	Nigeria	OQ418257	—	—	—
*Sphaerellopsis paraphysata*	PUR87214	*Uromyces unioniensis*	Brazil	OQ418262	OQ418163	OQ743703	—
*Sphaerellopsis paraphysata*	PUR88231	*Puccinia oahuensis*	Brazil	OQ418264	—	—	—
*Sphaerellopsis paraphysata*	PUR9003	*Catenulopsora praelonga*	USA	OQ418268	—	—	—
*Sphaerellopsis paraphysata*	PURF10798	*Crossopsora fici*	Uganda	OQ418275	—	—	—
*Sphaerellopsis paraphysata*	PURF11444	*Puccinia posadensis*	Trinidad	OQ418279	—	—	—
*Sphaerellopsis paraphysata*	PURF11445	*Puccinia polysora*	St. Vincent & the Grenadines	OQ418280	—	—	—
*Sphaerellopsis paraphysata*	PURF11502	*Puccinia purpurea*	Venezuela	OQ418282	—	—	—
*Sphaerellopsis paraphysata*	PURF12985	*Uromyces costaricensis*	Trinidad	OQ418286	—	—	—
*Sphaerellopsis paraphysata*	PURF14478	*Phakopsora clemensiae*	India	OQ418287	OQ418169	OQ743707	—
*Sphaerellopsis paraphysata*	PURF14635	*Puccinia nakanishikii*	India	OQ418288	—	—	—
*Sphaerellopsis paraphysata*	PURF14757	*Phakopsora africana*	Uganda	OQ418290	—	—	—
*Sphaerellopsis paraphysata*	PURF14933	*Puccinia eleocharidis*	Trinidad	OQ418291	—	—	—
*Sphaerellopsis paraphysata*	PURF14951	*Phakopsora loudetiae*	Kenya	OQ418292	—	—	—
*Sphaerellopsis paraphysata*	PURF15450	*Phakopsora pallescens*	Colombia	OQ418293	—	—	—
*Sphaerellopsis paraphysata*	PURF17943	*Puccinia duthiae*	India	OQ418301	—	—	—
*Sphaerellopsis paraphysata*	PURF18709c	*Uromyces manihotis*	Brazil	OQ418302	—	—	—
*Sphaerellopsis paraphysata*	PURF19059	*Uromyces costaricensis*	Brazil	OQ418304	—	OQ743710	—
*Sphaerellopsis paraphysata*	PURF19059-2	*Uromyces costaricensis*	Brazil	—	—	OQ743711	—
*Sphaerellopsis paraphysata*	PURF19505	*Uromyces linearis*	Nigeria	OQ418306	—	—	—
*Sphaerellopsis paraphysata*	PURF19703	*Puccinia cenchri*	Nigeria	OQ418307	—	—	—
*Sphaerellopsis paraphysata*	PURF19904	*Puccinia erythropus*	China	OQ418308	—	—	—
*Sphaerellopsis paraphysata*	PURF19927	*Puccinia paspalina*	China	OQ418309	—	—	—
*Sphaerellopsis paraphysata*	PURF19929	*Puccinia pogonatheri*	China	OQ418310	—	—	—
*Sphaerellopsis paraphysata*	PURF2490	*Puccinia infuscans*	Ecuador	OQ418314	—	—	—
*Sphaerellopsis paraphysata*	PURF2800	*Uromyces bonariensis*	Venezuela	OQ418315	—	—	—
*Sphaerellopsis paraphysata*	PURF4672	*Puccinia cacabata*	Brazil	OQ418325	—	—	—
*Sphaerellopsis paraphysata*	PURF4770	*Puccinia levis*	Bolivia	OQ418326	—	—	—
*Sphaerellopsis paraphysata*	PURF4897	*Uromyces setariae-italicae*	Trinidad	OQ418328	—	OQ743714	—
*Sphaerellopsis paraphysata*	PURF7972	*Puccinia neorotundata*	Peru	OQ418332	OQ418175	OQ743715	—
*Sphaerellopsis paraphysata*	PURF9841	*Puccinia thaliae*	Argentina	OQ418338	—	—	—
*Sphaerellopsis paraphysata*	PURN10179	*Puccinia minuta*	Brazil	OQ418339	—	—	—
*Sphaerellopsis paraphysata*	PURN10369	*Puccinia faceta*	Brazil	OQ418340	—	—	—
*Sphaerellopsis paraphysata*	PURN10826	*Kweilingia divina*	USA	OQ418341	OQ418178	OQ743718	—
*Sphaerellopsis paraphysata*	PURN10826_2	*Kweilingia divina*	USA	—	—	OQ743719	
*Sphaerellopsis paraphysata*	PURN10850	*Puccinia invenusta*	Guam	OQ418342	—	—	—
*Sphaerellopsis paraphysata*	PURN11077	*Kweilingia divina*	Taiwan	OQ418343	—	—	—
*Sphaerellopsis paraphysata*	PURN11077_2	*Kweilingia divina*	Taiwan	—	—	OQ743720	—
*Sphaerellopsis paraphysata*	PURN1120	*Mikronegeria fagi*	Argentina	OQ418344	—	—	—
*Sphaerellopsis paraphysata*	PURN11634 (PURP)	*Puccinia purpurea*	USA	OQ418348	OQ418181	OQ743723	OQ587606
*Sphaerellopsis paraphysata*	PURN15263	*Phakopsora* sp.	Taiwan	OQ418353	—	OQ743725	OQ587605
*Sphaerellopsis paraphysata*	PURN15329	*Uromyces setariae-italicae*	Bolivia	OQ418355	OQ418184	OQ743727	OQ587608
*Sphaerellopsis paraphysata*	PURN15342	*Uromyces hedysari-paniculati*	Guyana	—	OQ418185	OQ743728	—
*Sphaerellopsis paraphysata*	PURN15343	*Puccinia commelinae*	Guyana	OQ418356	—	—	—
*Sphaerellopsis paraphysata*	PURN15344	*Puccinia duthiae*	Guyana	OQ418357	OQ418186	OQ743729	—
*Sphaerellopsis paraphysata*	PURN15498	*Uromyces tenuicutis*	Guyana	OQ418358	—	—	—
*Sphaerellopsis paraphysata*	PURN15511	*Puccinia obliquo-septata*	Guyana	OQ418359	OQ418187	OQ743730	—
*Sphaerellopsis paraphysata*	PURN16553	*Puccinia obliquo-septata*	Guyana	OQ418364	OQ418191	OQ743732	—
*Sphaerellopsis paraphysata*	PURN16743	*Puccinia* sp.	Venezuela	OQ418365	OQ418192	OQ743733	—
*Sphaerellopsis paraphysata*	PURN22990	*Melampsora* sp.	China	OQ418368	—	—	—
*Sphaerellopsis paraphysata*	PURN2908	*Phakopsora rossmanii*	Brazil	OQ418374	—	—	—
*Sphaerellopsis paraphysata*	PURN4122	*Melampsora epitea*	Brazil	OQ418388	OQ418202	OQ743738	—
*Sphaerellopsis paraphysata*	PURN4123	*Melampsora epitea*	Brazil	OQ418389	OQ418203	—	—
*Sphaerellopsis paraphysata*	PURN5064	*Uromyces rhynchosporae*	Papua New Guinea	OQ418397	—	—	—
*Sphaerellopsis paraphysata*	PURN5574	*Puccinia stenotaphri*	Ecuador	OQ418400	—	—	—
*Sphaerellopsis paraphysata*	PURN5917	*Puccinia arachidis*	Brazil	OQ418401	—	—	—
*Sphaerellopsis paraphysata*	PURN9602	*Chaconia ingae*	Brazil	OQ418404	—	—	—
*Sphaerellopsis paraphysata*	TA427	*Puccinia cf. cyperi tegetiformis*	Benin	OQ418407	OQ418212	—	—
*Sphaerellopsis* sp.	PUR23925	*Puccinia montanensis*	USA	OQ418223	—	—	—

We successfully amplified 195 DNA sequences of *Sphaerellopsis* from ITS rDNA, 58 sequences from LSU rDNA, 48 from *tef1*, and eight from *rpb2*. Although we amplified the four loci for some *Sphaerellopsis* specimens, degradation of DNA in older specimens limited the ability to obtain complete locus datasets for many specimens. Nevertheless, we successfully amplified the ITS region of 163 specimens collected between 1883 and 1998. The oldest *Sphaerellopsis* specimen whose ITS region was successfully amplified was collected in 1883 on *Melampsora medusae* from the United States (voucher number: PUR2041, GenBank accession number: OQ418220). Lastly, we isolated a strain of *S. macroconidialis* (SP28) from freshly collected material, which was the basis of the interaction experiments between the conidia of *S. macroconidialis* and germinated urediniospores of *Puccinia polysora*, the host from which it was collected.

### Nucleotide alignment dataset and phylogenetic inferences

**Fig. 1:**
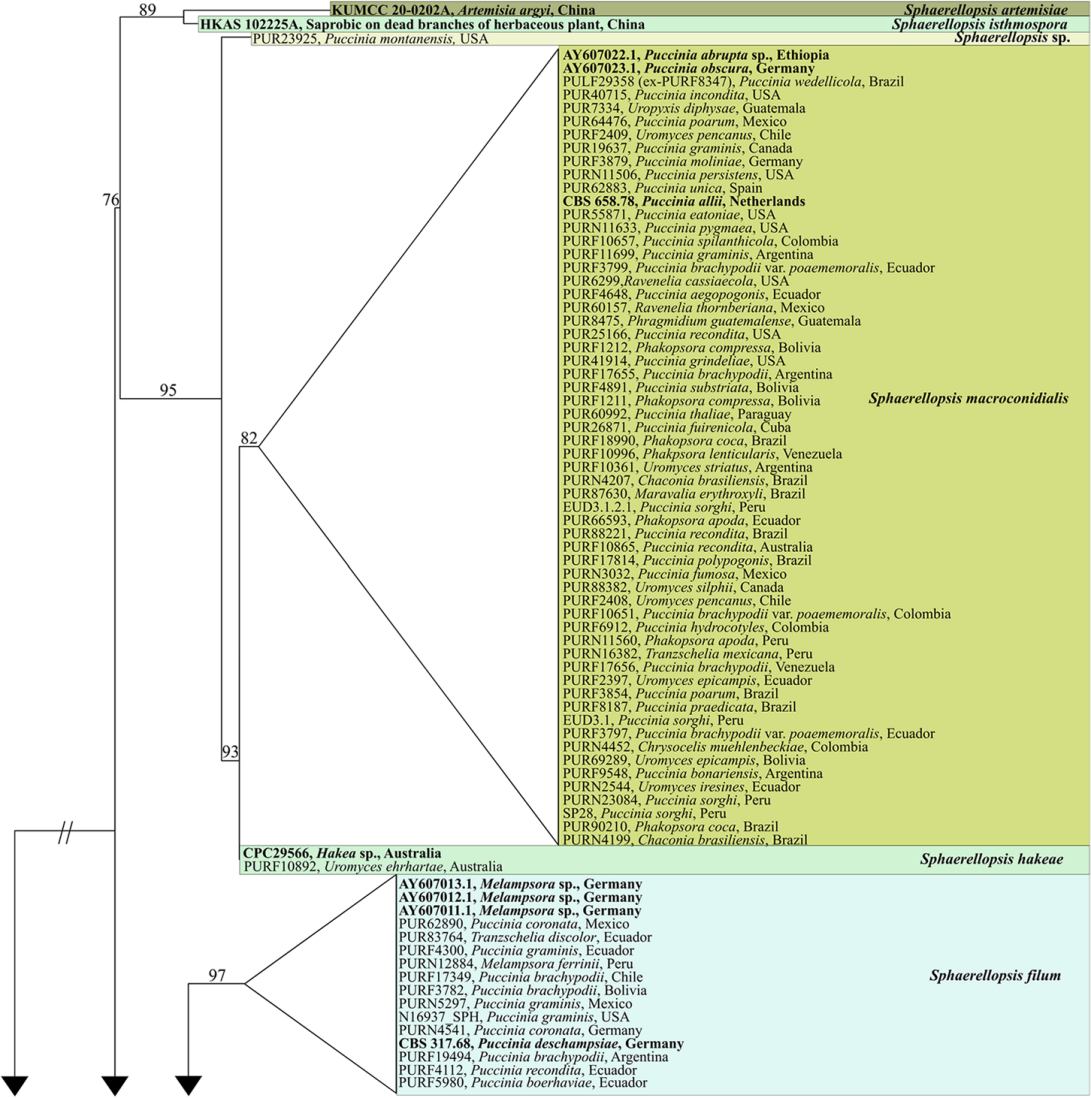

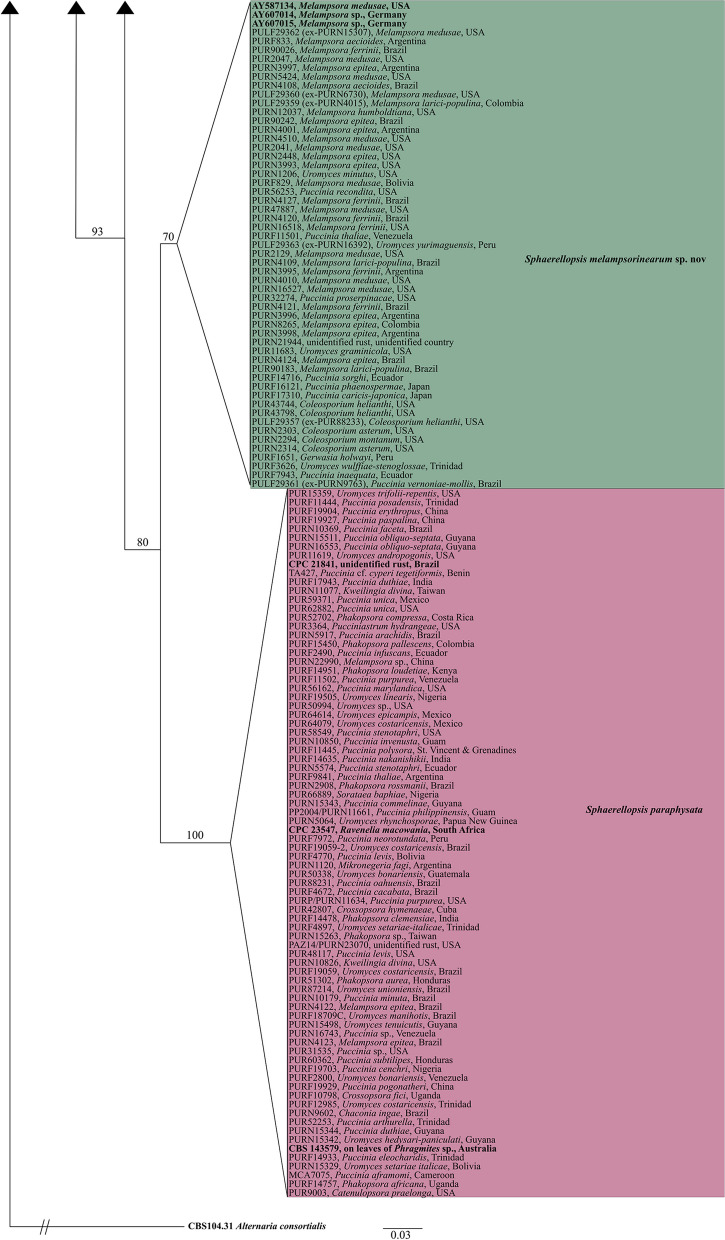
The top-scoring ML phylogenetic tree of the genus *Sphaerellopsis* reconstructed from the four-locus-concatenated dataset (ITS, LSU, *tef1*, and *rpb2*). The ML bootstrap value is presented above each branch. Colors delimit clades, each labeled with the corresponding *Sphaerellopsis* species. Taxa labels are written on the tree as “PUR voucher,” “the host rust where *Sphaerellopsis* was found,” and “the origin/locality of each specimen.” Reference sequences and outgroup taxa are written in bold. The tree was rooted to *Alternaria consortialis* CBS 104.31. Refer to supplementary figure S1. to see the fully resolved phylogram showing branch lengths and support values

Our multi-locus phylogenetic analysis consisted of a four-locus-concatenated dataset of 1996 characters, of which 352 were parsimony-informative. The percentage of parsimony-informative characters per gene region was 3.4% for ITS, 1.3% for LSU, 7.1% for *tef1*, and 5.8% for *rpb2*. We analyzed 219 individuals, of which 16 were sequences from previously identified *Sphaerellopsis* taxa, and *Alternaria consortialis* served as an outgroup taxon (Table 3). The following models were selected by ModelFinder (AICc): JC for ITS, GTR + F + R2 for LSU, TIM + F + G4 for *tef1*, and TIM2e + I for *rpb2*. Our maximum likelihood analysis revealed eight supported clades (Fig. 1, supplementary Figure S1), all of which have bootstrap support ≥ 70%.

### Species Diversity of *Sphaerellopsis* associated with rust fungi

Five species of *Sphaerellopsis* were recovered from our sampling: four of the seven previously accepted species, and one undescribed species (Fig. 1). *Sphaerellopsis paraphysata* was the most common species within our screened collections, found on 77 rust specimens, followed by *S. macroconidialis* on 56 and *S. filum* on 12. *Sphaerellopsis hakeae* was found in one rust specimen, and *S. artemisiae* and *S. isthmospora* were not found in this study. One species, *Sphaerellopsis anomala* is not represented in our analyses, due to lack of sequence data. Two other well-supported clades were found in the phylogeny that do not represent previously published *Sphaerellopsis* species. One of these consisted of a single specimen found on sori of *Puccinia montanensis* from the United States collected in 1896 (voucher number: PUR23925). We amplified the ITS region of this specimen and took macro photographs. However, due to the scarce and dry material, the specimen’s morphology and amplification of other gene regions were impossible. Thus, it is uncertain whether this represents an undescribed species. The other clade comprised 55 specimens from *Pucciniales* collected between 1883 and 2016, including a specimen that contained both the asexual and sexual morphs. This new species is described as *Sphaerellopsis melampsorinearum* sp. nov. below.

### Taxonomy

***Sphaerellopsis melampsorinearum*** **Gomez-Zap. & Aime, sp. nov.**

Figure 2.

**Fig. 2:**
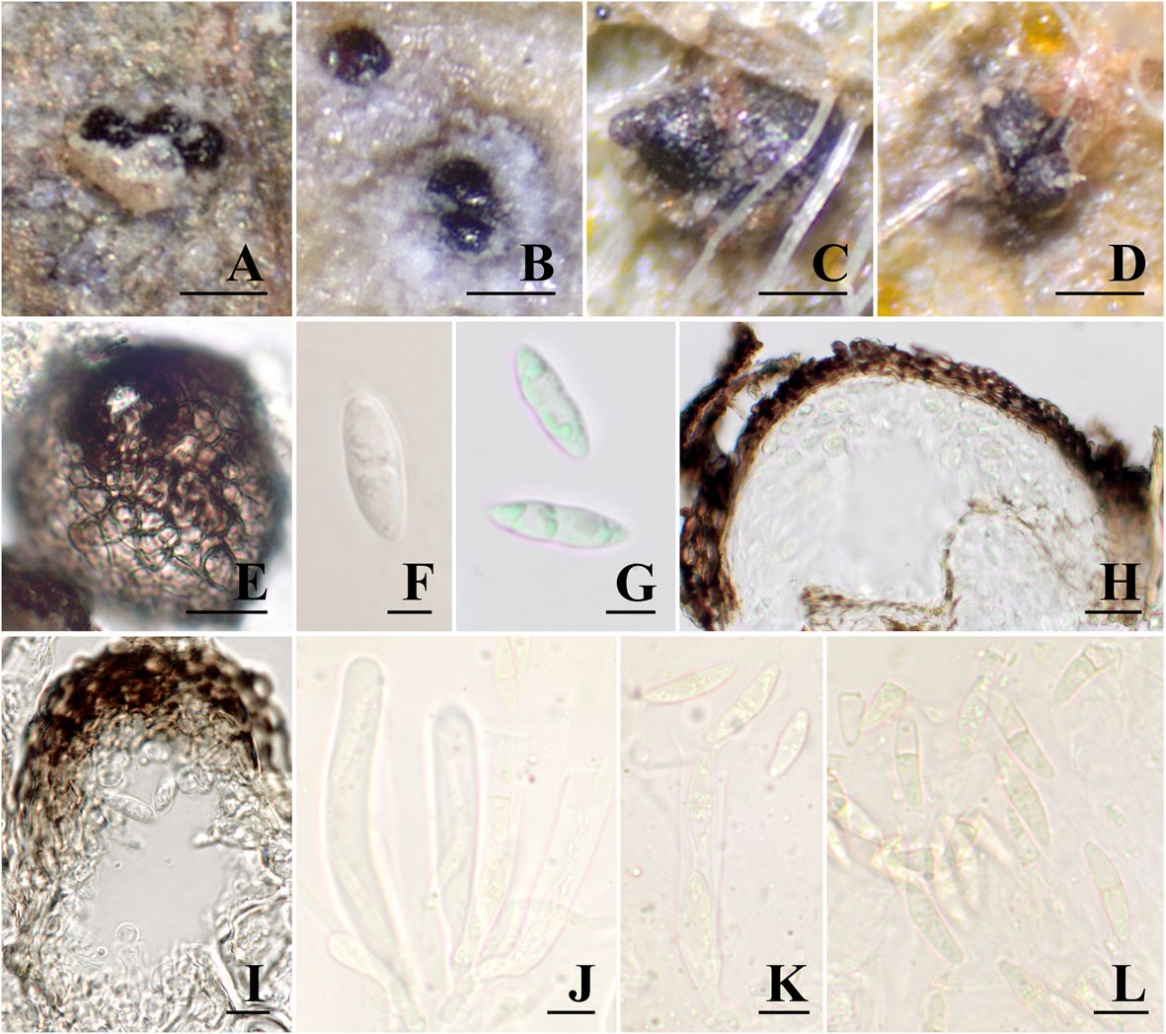
*Sphaerellopsis melampsorinearum* sp. nov. (PUL F29362, PUL 29,360, PUL F29361). **A**, **B** Conidiomata developed on sori. **C**, **D** Ascomata developed on sori. **E** Outer layers of conidioma, textura angularis. **F**, **G** Conidia. H Vertical section through ascomata. **I** Conidiogenous cells. **J**, **K **Asci and pseudoparaphyses. **K**, **L** Ascospores. Bars: a–d = 100 μm, e–g = 10 μm, h = 20 μm, i–l = 10 μm

Mycobank No: MB847464.

**Etymology: **Named after the large number of rust hosts that belong to the suborder *Melampsorineae*.

**Diagnosis: **Similar to *S. filum* but differs in conidiomata size (up to 107 μm diam.), and conidia length [(9.1–)10.3–14.3(–16.6) µm].

**Type: Holotype**: United States, Indiana, Tippecanoe County, West Lafayette, on urediniospores of *Melampsora medusae* infecting *Populus deltoides*, 19 September 2015, M. Catherine Aime, s.n. (PUL F29362 (ex-PURN15307); GenBank accessions ITS–OQ418354, LSU–OQ418183, *tef1–O*Q743726, *rpb2–O*Q587607).

**Description**: Asexual morph–conidiomata associated with rust sori, pycnidial, erumpent, aggregated, globose, 48–107 μm, with central ostiole, outer layers dark brown cells textura angularis, 3.8–6.92 μm diam. Paraphyses not observed. Conidiophores reduced to conidiogenous cells. Conidiogenous cells line the inner cavity and are smooth, hyaline, globose to ampulliform. Conidia fusoid, hyaline, smooth, guttulate, 1-septate, slightly constricted at the septum, apex subobtuse, tapering to truncate hilum, (9.1–)10.3–14.3(–16.6) × (3–5) µm. Sexual morph–ascomata associated with rust sori, 76–162 μm diam., solitary or gregarious; loci immersed, brown in outer zone consisting of two to three rows of dark cells, hyaline in inner part, subglobose to ampulliform, with protruding papillate neck and ostiole. Pseudoparaphyses are filiform, septate, hyaline. Asci numerous, 8-spored, bitunicate, cylindrical-clavate, short stipitate, 59.6–101.3 × 8.5–10.4 μm. Ascospores irregularly biseriate, fusiform, hyaline to pale yellow, 15.2–21.3 × 3.8–6.0, 1-septate, slightly constricted at the septum, surrounded by a mucous sheath not easily perceived.

**Substrate/Host**: on rust sori of several rust species, principally species of the genus *Melampsora*, but also known to infect *Coleosporium* spp., *Puccinia* spp., *Uromyces* spp., and *Gerwasia holwayi.*

**Distribution**: Argentina, Bolivia, Brazil, Colombia, Ecuador, Germany, Japan, Peru, Trinidad, the continental United States of America, Venezuela.

**Additional materials examined: Brazil**, São Paulo, on urediniospores of *Puccinia vernoniae*-*mollis* infecting leaves of *Vernonia* sp., 17 February 1989, Anibal de Carvalho 89 − 7, containing teleomorph, (PUL F29361 (ex-PURN9763); GenBank accessions: ITS–OQ418405; LSU– OQ418210). **Colombia**, Antioquia, on urediniospores of *Melampsora larici*-*populina* infecting *Populus nigra*, 20 March 1989, V.M Pardo-Cardona s.n. (PUL F29359 (ex-PURN4015); GenBank accessions: ITS–OQ418383; LSU–OQ418199). **Peru**, Ucayali, on urediniospores of *Uromyces yurimaguasensis*, 22 October 2016, M. Catherine Aime MCA6471. (PUL F29363 (ex-PURN16392); GenBank accessions: ITS–OQ418361; LSU–OQ418189). **United States of America**, Illinois, on urediniospores of *Melampsora* sp. infecting *Populus* sp., 22 September 2012, M. Catherine Aime MCA5030 (PUL 29,360 (ex-PURN6730); GenBank accessions: ITS– OQ418402, LSU–OQ418209, *tef1–O*Q743742); Georgia, on urediniospores of *Coleosporium helianthi* infecting *Silphium compositum*, 24 August 1977, Yoshitaka Ono, John McCain & Joe F. Hennen 10,185 (PUL F29357 (ex-PUR88233); GenBank accession: ITS–OQ418265).

**Notes.** The conidiomata and length of conidia of *S. melampsorinearum* are smaller than for any other described *Sphaerellopsis* species. However, the width of the conidia of *S. melampsorinearum* is similar to *S. anomala*, *S. filum*, and *S. macroconidialis*. *Sphaerellopsis melampsorinearum* is distributed worldwide and infects a range of rust species in the *Pucciniaceae*, *Phragmidiaceae*, *Melampsoraceae*, and *Coleosporiaceae*. However, 41 out of 55 hosts rust hosts of *S. melampsorinearum* belong to suborder *Melampsorineae*.

### The sexual morph of *S. macroconidialis*

**Fig. 3:**
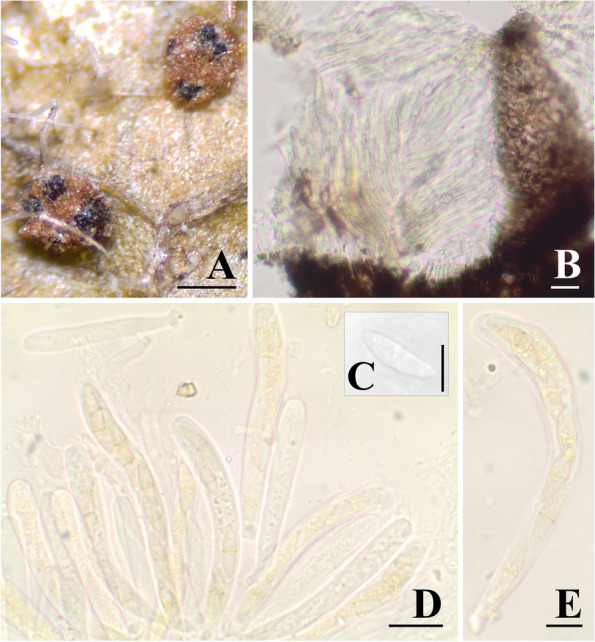
Teleomorph of *S. macroconidialis* (PUL F29358). **A** Ascomata. **B** Vertical section through ascomata. **C** Ascospore. **D** Asci and pseudoparaphyses. **E** Asci and ascospores. Bars: a = 200 μm, b, c = 20 μm, d, e = 10 μm

*Sphaerellopsis macroconidialis* is known from the asexual morph, and no sexual morph has been described. However, in this study, we recovered one specimen containing the sexual morph of *S. macroconidialis* (Fig. 3). This specimen was found in Brazil, Rio de Janeiro, associated with telia of *Puccinia wedeliicola* infecting the host plant *Wedelia trichostephia*, collected on 7 May 1922, by E.W.D Holway, #1822, (PUL F29358 (ex-PURF8347)) (Fig. 3). The ITS sequence obtained from this specimen shared 100% identity (239/239 no gaps) with *S. macroconidialis* CBS 233.51 (GenBank Accession No. MH856836.1). Morphology of the sexual morph is as follows: ascomata developing on rust sori, up to 123 μm diam., brown in outer zone, cells textura parenchymatic, hyaline in inner part, erumpent, gregarious; loci subglobose to ampulliform. Pseudoparaphyses are filiform, septate, hyaline. Asci are numerous, 8-spored, bitunicate, cylindrical-clavate, short stipitate, 68.2–106.7 × 7.3–11.1 μm. Ascospores are irregularly biseriate, fusiform, hyaline to pale yellow, 17.2–23 × 4.8–6.0, 1-septate, slightly constricted at the septum, surrounded by a hyaline mucous sheath not easily perceived.

### In-vitro interaction test between *S. macroconidialis* and *P. polysora *

**Fig. 4:**
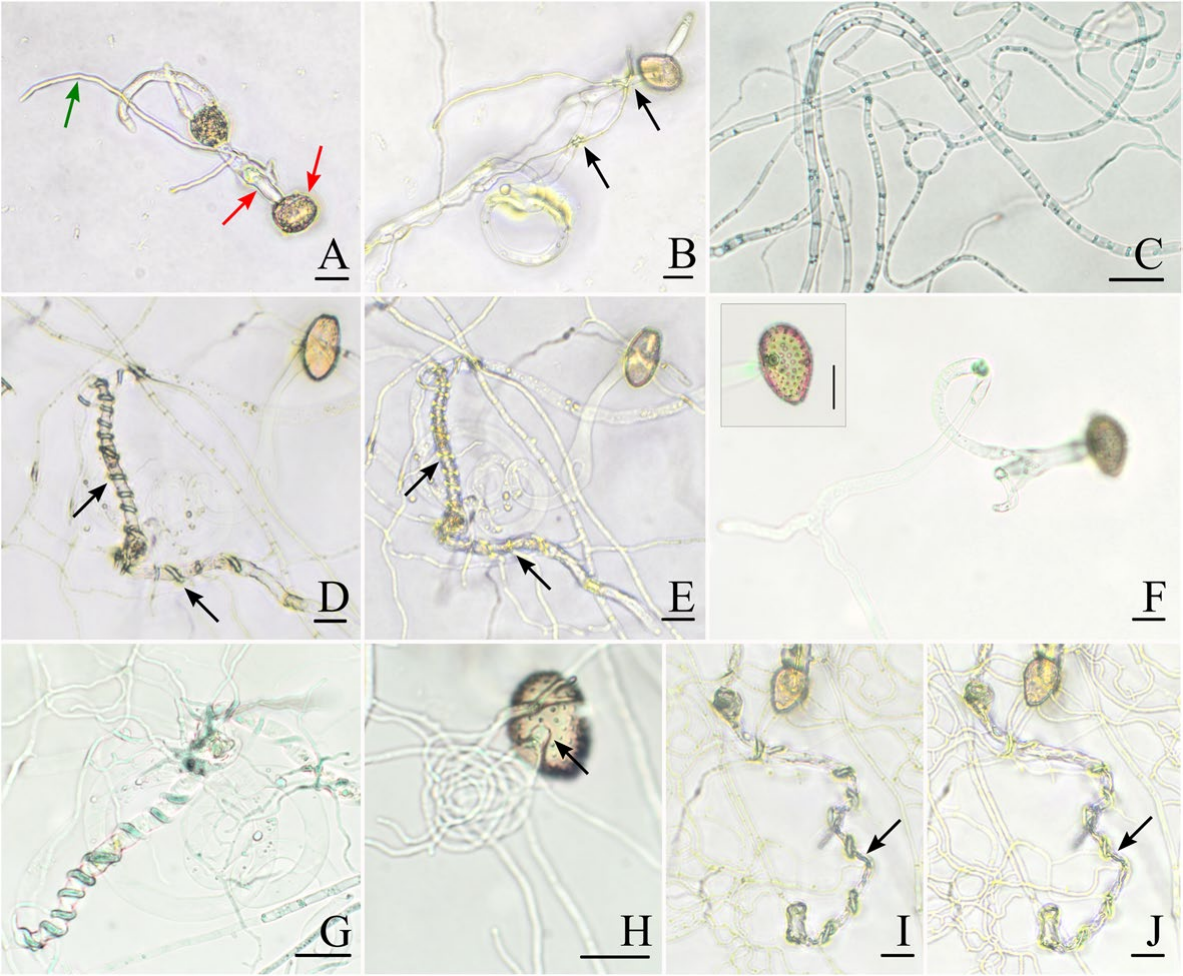
Light micrographs of *Sphaerellopsis macroconidialis* interacting with germinated urediniospores of *Puccinia polysora* *in-vitro*. A─B Day one after co-cultivation. A Red arrows point to the urediniospore and its germ tube, and the green arrow points to S. macroconidialis*S. macroconidialis* hypha. B Black arrows point to the first contact. C Negative control, hyphae of *S*. *macroconidialis *alone on day 12. D─E Day five after co-cultivation. Hyphae of *S. macroconidialis* form coils and tightly encircle germ tubes of *P. polysora*. Black arrows point to dense coils. F Negative control, urediniospores, and germ tubes alone on day 12. G─J Day 12 after co-cultivation. G Dense coils around a germ tube of *P. polysora*. H An appressorium (arrow) attached to the urediniospore. I and J Loss of turgor of germ tube of *P. polysora*. Scale bars: A-B, D-J = 20 μm, C = 50 μm

The interaction test of this study confirms the mycoparasitic strategy of *S. macroconidialis* on rust fungi. Five days after the co-cultivation, we observed hyphae of *S. macroconidialis* growing along the germ tubes of *P. polysora* and coiling around them (Fig.4).

The germ tubes of *P. polysora* measured 6.5 μm in diam., while those of *S. macroconidialis* measured 1.8 μm in diam., making them easy to distinguish. During the first day after co-cultivation, we observed the first contact between germinated conidia of *S. macroconidialis* and germ tubes of urediniospores of *P. polysora*. Then, during the next four days, hyphae of *S. macroconidialis* started to grow over the urediniospores and their germ tubes, but without clear evidence of antagonism. However, on the fifth day of co-cultivation, *S. macroconidialis* began coiling around rust germ hyphae. Coils tightly encircled the germ tubes. However, the cell wall of the germ tubes was not disrupted. Such coils were not seen on *S. macroconidialis* hyphae inoculated alone. During the next six days, we did not notice any new sign of mycoparasitic mechanism against *P. polysora*. Nonetheless, on day 12, we noticed the formation of an appressorium attached to a urediniospore and turgor loss of a few germ tubes already coiled by *S. macroconidialis*. Loss of turgor was not seen on germinated urediniospores inoculated alone. After 12 days of observations, *S. macroconidialis* hyphae grew abundantly, and no other antagonistic events could be observed.

## Discussion

Characterization studies of fungi with potential as BCAs are essential to the development of applied microbial biocontrol of plant diseases. Although the fungal genus *Sphaerellopsis* is commonly considered a rust mycoparasite due to its association with several rust species, studies of this genus are scarce, and its biocontrol potential is unknown. To evaluate *Sphaerellopsis* as a candidate BCA, we screened thousands of rust collections for the presence of *Sphaerellopsis* (Supp. Table 1). We generated sequence data for nearly 200 *Sphaerellopsis* specimens found on rust fungi collections at four loci, including the ITS, which has previously been shown as a good barcoding region for *Sphaerellopsis* species (Trakunyingcharoen et al. 2014) and three other loci—LSU, *rpb2*, and *tef1* for phylogenetic resolution (Fig. 1). We then use these data to characterize various aspects of *Sphaerellopsis* biology including species diversity, geographic distribution, and host specificity. Finally, we examined the interactions between *S. macroconidialis* and *Puccinia polysora* to infer initial infection strategies. These results can help determine the suitability of the application based on the BCA’s location and mode of action.

### *Sphaerellopsis* species frequencies

*Sphaerellopsis macroconidialis* and *S. paraphysata* were the most common species associated with rust fungi in this study. *S. macroconidialis* was found on species in ten rust genera, and *S. paraphysata* on 12 rust genera (Fig. 1). Contrary to expectations, the type species, *S. filum*, previously reported from 30 rust genera and 369 rust species (Kranz and Brandenburger 1981), was not frequently collected. We found *S. filum* associated with only three rust genera: *Melampsora*, *Puccinia*, and *Tranzschelia.* As prior studies have shown, other species of *Sphaerellopsis* were frequently misidentified as *S. filum* in the past (Trakunyingcharoen et al. 2014), which could explain the discrepancy.

### Host-specificity of  *Pucciniales*-infecting *Sphaerellopsis* species

Prior studies have found host-specificity in species of *Sphaerellopsis* (Liesebach and Zaspel 2004), Nischwitz et al. 2005), Kajamuhan et al. 2015). In contrast, our study does not show any signature of host-specificity for the *Sphaerellopsis* species analyzed (Fig. 1). For example, *S. macroconidialis* was found to be associated with species from several genera across the rust tree of life (Aime and McTaggart 2020) including *Chaconia*, *Phakopsora*, *Phragmidium*, *Puccinia*, *Ravenelia*, and *Uropyxis*, among others (Fig. 1). Similarly, *S. paraphysata* was associated with species from multiple rust genera including *Crossopsora*, *Kweilingia*, *Melampsora*, *Mikronegeria*, *Phakopsora*, *Puccinia*, *Sorataea*, and *Uromyces*, among others. *Sphaerellopsis filum* was recovered infecting hosts from three suborders of *Pucciniales*; and *S. melampsorinearum* was found on four families of *Pucciniales*, with the majority of hosts within the subphylum *Melampsorineae*.

Differences between our and previous work are likely due to limited sampling in prior studies, which only examined *Sphaerellopsis* species associated with *Puccinia* species on grass hosts and *Melampsora* species on poplars. The dataset of Liesebach and Zaspel (2004) and Nischwitz et al. (2005) did not exceed 20 isolates, and the sampling of Kajamuhan et al. (2015) comprised 82 isolates collected from *Puccinia* species. In contrast, our dataset covered 19 rust genera and 216 specimens. Although *S. paraphysata* and *S. macroconidialis* are predominantly associated with *Puccinia* specimens and *S. melampsorinearum* with *Melampsora* specimens, both are also associated with rust species from other genera. Likewise, our data do not show preference of *Sphaerellopsis* species for rusts at even the family rank. For example, *S. paraphysata* included rust hosts in the families *Melampsoraceae*, *Phakopsoraceae*, and *Pucciniaceae*, which span three different rust subphyla.

Interestingly, we found *Sphaerellopsis* species infecting rusts on many economically important hosts such as maize, wheat, and poplars. However, we did not find any *Sphaerellopsis* infecting *Hemileia vastatrix*, the causal agent of coffee leaf rust, despite examination of 42 specimens of this rust collected from throughout its range. Nor does *Hemileia vastatrix* appear on prior lists of *Sphaerellopsis* rust hosts (Kranz and Brandenburger 1981). Keener (1934) suggested that a possible limiting factor of *Sphaerellopsis* infection could be the type of sorus produced. *Hemileia* species, for example, form suprastomatal sori that protrude through the stoma like a “bouquet” (McCain 1983) and do not tear the epidermis of the host plant. In addition to *Hemileia*, we also screened other rust specimens of the family *Zaghouaniaceae* that form suprastomatal sori; all were also free of *Sphaerellopsis* (Supplementary Table 1). Only one specimen of *Mikronegeria fagi* was found with associated black fruiting bodies resembling *Sphaerellopsis*. However, due to the scarcity and age of this particular specimen, we were unable to confirm it as a species of *Sphaerellopsis*. Thus, it remains inconclusive, but likely, that *Sphaerellopsis* species are restricted to infecting hosts that do not form suprastomatal sori.

### Geographic distribution

**Fig. 5:**
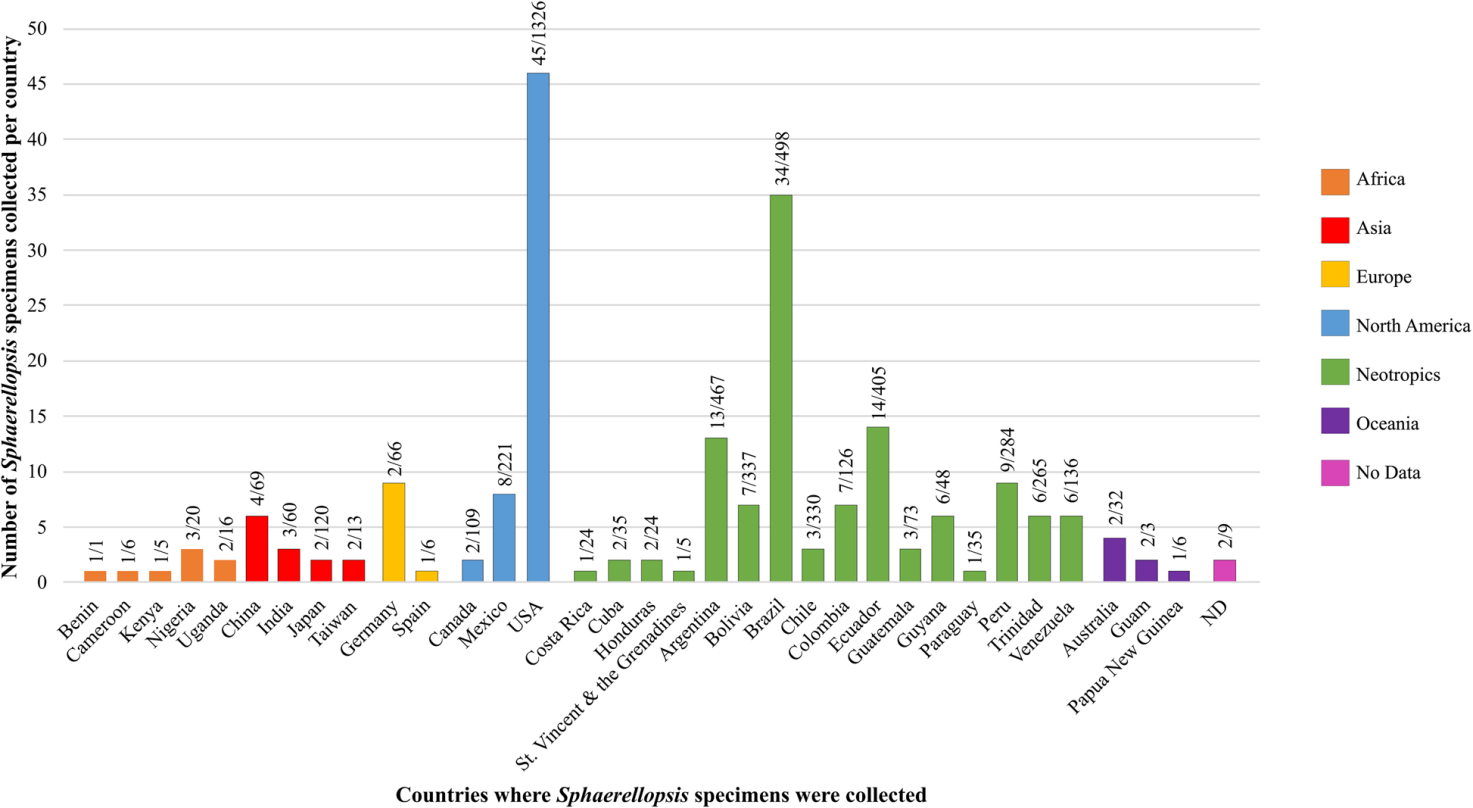
Origin/localities of the confirmed *Sphaerellopsis* specimens associated with rust fungi. The numerator above the bar indicates the number of *Sphaerellopsis* specimens collected per country; the denominator indicates the total rust specimens screened at PUR for the presence of *Sphaerellopsis* per country. Countries are colored by geographic regions

**Fig. 6:**
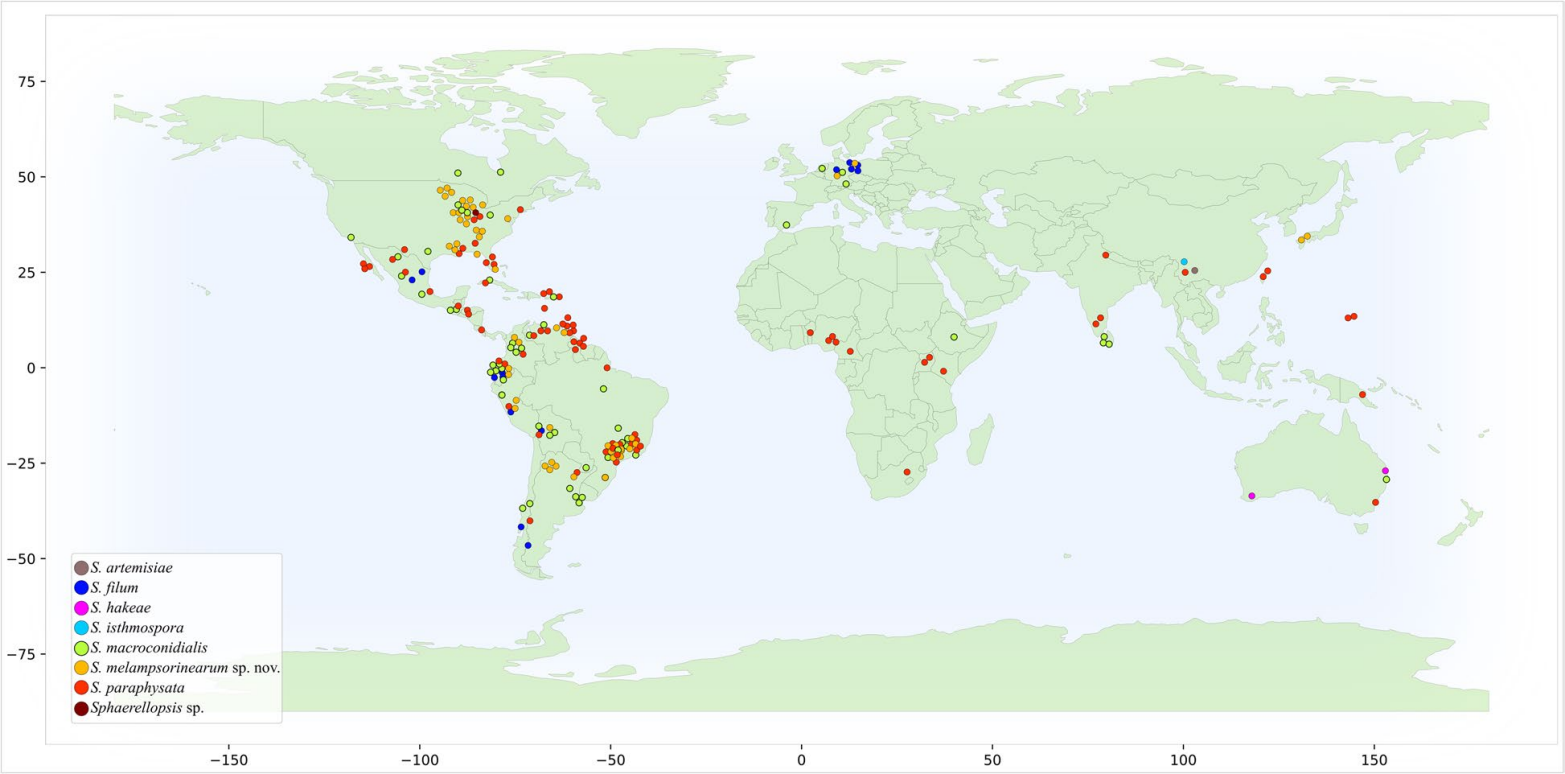
Geographical distribution of *Sphaerellopsis* specimens examined in this study. Each circle represents one specimen, and each color represents one *Sphaerellopsis* species

This study included *Sphaerellopsis* specimens associated with *Pucciniales* collected in 34 countries across the globe. Eight specimens were from Africa, 11 from Asia, three from Europe, 55 from North America, 115 from the Neotropics, and five from Oceania (Fig. 5; Table 3). Our results suggest that *S. macroconidialis, S. paraphysata, S. filum*, and *S. melampsorinearum* have a cosmopolitan distribution and are adapted to different environmental conditions in both temperate and tropical regions (Fig. 6). However, *S. paraphysata* appears to be more abundant in the tropics. *Sphaerellopsis hakeae* may be an exception to this pattern, as both specimens of this species analyzed were from Australia, from where it was also described (Crous et al. 2016). The small sample size in our study limits any conclusive inferences, but it is worth noting that this species was not recovered even among the other Oceania specimens examined. Similarly, our study did not recover any additional specimens of *S. artemisiae* or *S. isthmospora*, both currently only known from China (Doilom et al. 2021; Phookamsak et al. 2019).

The dispersal biology of *Sphaerellopsis* species is not well studied. Kuhlman et al. (1978) hypothesized that conidia of *Sphaerellopsis* did not disperse over long distances but rather spread locally via water splashing to nearby hosts. Our results would suggest that *Sphaerellopsis* could also be capable of long-distance dispersal. Rust spores can be dispersed through wind currents and may cross continents, and it is possible that the much smaller conidia of *Sphaerellopsis* species may be passively dispersed along with their much larger host spores. However, further studies in the dispersion mode of *Sphaerellopsis* are necessary to support this hypothesis.

### Sexual morphs of *Sphaerellopsis*

*Eudarluca* has been considered the sexual morph of *Sphaerellopsis*. Because these are congeneric (Keener 1951; Yuan et al. 1998), *Sphaerellopsis*, the older name, has priority for these fungi. The genus *Eudarluca* was erected in 1908 by Spegazzini to place “a new pyrenomycete” associated with the uredosori of an unknown rust, infecting *Canna* sp. in the Botanical Garden in São Paulo, Brazil (Spegazzini 1908). Spegazzini subsequently named *Eudarluca australis* as the type species of the genus. However, later in 1966, Eriksson combined several species with *E. australis* into *E. caricis* based on an overview of the taxonomy, nomenclature, and ecology of *E. caricis* (Eriksson 1966). The specific epithet “caricis” was kept based on the basionym *Sphaeria caricis* described by Fries in 1823 (Fries 1823). The original specimen of *Sphaeria caricis* was collected from uredinia of a rust species on *Carex* spp. Since *Sphaerellopsis filum* was misapplied in the past as the most common species associated with rust fungi, it was thought to be congeneric with *E. caricis* (Yuan et al. 1998), a position that was not supported by the detailed analyses of Trakunyingcharoen et al. (2014). While we were able to identify the sexual morph of *S. macroconidialis* on telia of *Puccinia wedeliicola* infecting the host plant *Wedelia trichostephia*, and the sexual morph of *S. melampsorinearum* on uredinia of *Puccinia vernoniae-mollis* on the leaves of *Vernonia* sp., we were unsuccessful in locating a sexual morph of a *Sphaerellopsis* specimen that would be consistent with *E. caricis*, and thus the asexual morph and correct name for this species remains unknown.

The two sexual specimens of *Sphaerellopsis* described in this study (Figs. 2 and 3) were collected in the Neotropics. Eriksson (1966), Ramakrishnan and Narasimhalu (1941), and Sebesta (1963) found that high humidity, such as is found in the tropics, favored production of the sexual morph in *Sphaerellopsis*. Similarly, when Västerbotten found the teleomorphic state of *Sphaerellopsis* in the Summer of 1962 in northern Sweden, the locality was a hollow in a compost heap, a few meters from a rivulet giving microclimate conditions “similar to the tropics” (Eriksson 1966). The host plant may also play a role in development of the sexual morph of *Sphaerellopsis*. For example, Eriksson (1966) noted that the sexual morph was most commonly found on plants in Poaceae and Cyperaceae due to their continuous growth and ability to form high-humidity microclimates. Although this hypothesis has not yet been experimentally tested, our findings are consistent with a high humidity requirement for sexual morph development.

### *Sphaerellopsis* infection strategies and antagonism between *S. macroconidialis *and *P. polysora*

The two earliest diverging species in our analyses, *S. artemisiae* and *S. isthmospora*, were not recovered on any rust samples in our study and are likely not associated with *Pucciniales* (Fig. 1). It has been posited that several trophic strategies ranging from mycoparasitism to saprotrophism to plant pathogen may have evolved within *Sphaerellopsis* (e.g., Hulea 1939; Eriksson 1966). Nicolas and Villanueva (1965) posited that the anamorph of *Sphaerellopsis* species might be able to utilize a large number of carbon compounds; Eriksson (1966) hypothesized that *Sphaerellopsis* species might feed on plant tissue but that other factors, such as specific compounds secreted from the rust, might be required for *Sphaerellopsis* to develop its fruiting bodies. Our data would support a hypothesis of an original plant-associated trophic strategy for members of this genus, that later transitioned to a mycoparasitic strategy on plant pathogenic rusts.

This study confirms *S. macroconidialis* as a mycoparasite of rust fungi. Coiling and appressorium formation by *S. macroconidialis* and turgor loss of germ tubes of *P. polysora* are evident signs of antagonistic relationships between these two fungi (Fig. 4). Appressorium formation and coiling are the most common mechanisms of mycoparasites to attack their host pathogens. For example, *Trichoderma harzianum* and *Trichoderma atroviride* show the same mechanism, coiling around its host, *Rhizoctonia solani*, and forming appressoria as an early event before hyphal damage (Benhamou and Chet 1993; Benítez et al. 2004; Chet et al. 1981; Rocha-Ramírez et al. 2002). Similarly, *Simplicillium lanosoniveum* and *Cladosporium tenuissimum* form appressoria and helix-shaped hyphae around urediniospores of the soybean rust *Phakopsora pachyrhizi* (Ward et al. 2011), and aeciospores of the two-needle pine stem rusts *Cronartium flaccidum*, and *Peridermium pini* (Moricca et al. 2001), respectively. *Sphaerellopsis paraphysata* has also been found coiling around urediniospores of *Puccinia substriata*, but appressorium formation was not seen in this study (Anandakumar et al. 2019).

The formation of helix-shaped hyphae of mycoparasites around the structures of their fungal hosts is a phenomenon usually dependent on lectin recognition. Fungal lectins are carbohydrate-binding proteins located on the fungal surface, which play a role in the recognition and defense of other organisms (Lebreton et al. 2021). Once the mycoparasite recognizes the lectins of the fungal host upon first physical contact, the mycoparasite hyphae start coiling around the fungal host for colonization and further infection (Omann and Zeilinger 2010). Thus, since *S. macroconidialis* was observed coiling around germ tubes of *P. polysora* on day five after co-cultivation, genes coding for lectins-binding proteins might be up-regulated during the first four days. Many lectins have been identified in filamentous fungi and yeasts (Lebreton et al. 2021), but information on these proteins in rust fungi is scarce.

Although we observed appressorium formation by *S. macroconidialis* in the interaction test, these were rare. We only observed one appressorium-like structure attached to a urediniospore on day 12 of co-cultivation (Fig. 4 H). This appressorium was not formed over the germ pore of the urediniospore, and the spore showed no signs of turgor loss. Because we stopped our observations on day 12 due to the overgrowth of *S. macroconidialis* hyphae, it is impossible to know if the appressorium had any mycoparasitic effect on the rust spore. Appressorium formation was also not observed on *Sphaerellopsis paraphysata* infecting urediniospores of *P. substriata* previously (Anandakumar et al. 2019). Given the late appearance of appressoria, *Sphaerellopsis* likely do not utilize these as the primary mode for penetrating rust fungi. In contrast, *Sphaerellopsis* species are likely to secrete lytic enzymes (e.g., chitinases, glucanases, and proteases) to infect host rusts once their hyphae coiled around rust structures. We noticed this effect on day 12, where some germ tubes of *P. polysora* lost turgor (Fig. 4 I, J). Although we did not conduct studies to detect enzymatic secretion, our experimental design may be helpful for future secretome analyses.

## Conclusion

In this study we attempted to fill some of the knowledge gaps surrounding *Sphaerellopsis*, with emphasis on obtaining data that would help to evaluate species as potential biological control agents for diseases caused by rust fungi. We demonstrate that *Sphaerellopsis* species are widespread and often incidentally co-collected with their rust hosts. Therefore, herbarium specimens may provide a rich source of data about these fungi. Also, *Sphaerellopsis* species do not appear to be specific to their rust hosts, in general, although there is a signal that some species may be climatically adapted. One new species recovered from herbarium specimens was described, *S. melampsorinearum,* and  the sexual morph of *S. macroconidialis *was characterized. Finally, we confirmed that mycoparasitic strategy of *S. macroconidialis* on *P. polysora*.

### Supplementary Information

Supplementary Material 1.

Supplementary Material 2.

Supplementary Material 3.

## Data Availability

All data generated or analyzed during this study are included in this published article [and its supplementary information files].

## Abbreviations

AICc: Akaike's information criterion corrected
BLAST: Basic Local Alignment Search Tool
EF1a: Elongation factor 1-alpha gene
ITS: Ribosomal DNA internal transcribed spacer region
LSU: Ribosomal DNA large subunit region
ML: Maximum likelihood phylogenetic analysis
MUSCLE: Multiple Sequence Comparison by Log- Expectation
NCBI: National Center for Biotechnology Information
PCR: Polymerase Chain Reaction
RPB2: DNA-directed RNA polymerase II subunit 2 gene
